# Can an HLB-resistant interstock block the long-distance movement of ‘*Candidatus* Liberibacter asiaticus’ within citrus trees?

**DOI:** 10.3389/fpls.2026.1733981

**Published:** 2026-03-25

**Authors:** Josiane C. Darolt, Laudecir L. Raiol-Junior, Everton V. Carvalho, Mônica N. Alves, Nelson A. Wulff, Jémima Bagéa, Olivier Gros, Bárbara Hufnagel, Raphael Morillon, Leandro Peña, Eduardo A. Girardi

**Affiliations:** 1 Fund for Citrus Protection, Araraquara, São Paulo, Brazil; 2 Embrapa Cassava and Fruits, Cruz das Almas, Bahia, Brazil; 3 Unité Mixte de Recherche Amélioration Génétique et Adaptation des Plantes Méditerranéennes et Tropicales (UMR AGAP) Institut, Univ. Montpellier, Centre de coopération International en Recherche Agronomique pour le Développement (CIRAD), Institut National de Recherche pour l’Agriculture, l’Alimentation et l’Environnement (INRAE), Institut Agro, Montpellier, France; 4 Institut de Systématique, Evolution, Biodiversité (ISYEB), Muséum National d’Histoire Naturelle, CNRS, Sorbonne Université, EPHE, Université des Antilles, Pointe-à-Pitre, France; 5 Instituto de Biología Molecular y Celular de Plantas (IBMCP), Consejo Superior de Investigaciones Científicas (CSIC) Polytechnic University of Valencia (UPV), Valencia, Spain

**Keywords:** *Citrus* spp., *C*Las movement, grafting, Huanglongbing, oceanian limes, resistance

## Abstract

Among citrus diseases, Huanglongbing (HLB) is recognized as the most destructive and economically damaging worldwide. It is mainly associated with ‘*Candidatus* Liberibacter asiaticus’ (*C*Las) being transmitted by *Diaphorina citri*. There are no curative treatments or commercial citrus varieties resistant to *C*Las. Wild Aurantioideae species have been widely screened and, recently, Oceanian genotypes graft- and sexually compatible with *Citrus* were identified as HLB-resistant; however, there is no information regarding their use as interstocks of commercial varieties yet. Under greenhouse conditions, six HLB-resistant genotypes were evaluated as interstocks between ‘Valencia’ sweet orange scion and ‘Rangpur’ lime rootstock, both susceptible to HLB, with ‘Valencia’ interstock as the control. Rootstocks were nucellar seedlings, and the scion was a commercial accession preimmunized with a mild CTV strain. Plants were assessed for *C*Las infection and titer in leaves, stem bark, and roots up to 12–24 months after graft-inoculation in the scion and compared to non-inoculated controls. Furthermore, molecular, anatomical, and biometric variables were investigated. As expected, the scion variety was colonized by *C*Las regardless of the genotype evaluated as interstock. Although bacteria were detected in the roots of most *C*Las-inoculated plants, *C*Las movement from the scion to the roots was blocked in 42% and 86% of composite plants when using a F1 hybrid of *C. australis* × *C. inodora* or an admixture hybrid of *C. glauca*, *C. australis*, and *C. australasica* as interstocks. Overall, *C*Las titers were similar in infected plant tissues among the evaluated genotypes, but titers were lower in interstock bark tissues compared to scion and rootstock ones. After one-two years of *C*Las + CTV infection (experiments I and II, respectively), the dry weight of the root system decreased by 50% in infected trees compared to control trees for most genotypes, and *C*Las + CTV infection was associated with changes in the sieve phloem and gene expression. These findings suggest that, despite CTV infection, interstocks derived from some hybrids of Australian citrus types have the potential to restrict the movement of *C*Las from the scion into the roots of infected citrus trees. Long-term evaluation of composite plants in field conditions is necessary to assess tree performance and, ultimately, the impact of *C*Las blockage by interstocking on HLB disease damage.

## Introduction

Huanglongbing (HLB) is the most important disease affecting the citrus industry worldwide. Since its report in the two main sweet orange juice-producing regions, São Paulo, Brazil, in 2004 and Florida, USA, in 2005 (Coletta Filho et al., 2004; Halbert, 2005), the sustainability of commercial citrus production in both areas has been threatened by the economic losses and high costs related to the spread and management of HLB (Graham et al., 2020; Alquézar et al., 2022). The disease-associated agents are phloem-restricted Gram-negative bacteria belonging to the group of α-proteobacteria of the genus ‘*Candidatus* Liberibacter spp (Lafléche and Bové, 1970; Jagoueix et al., 1994). In Brazil and the USA, ‘*Candidatus* Liberibacter asiaticus*’* (*C*Las) is the most destructive and prevalent bacterium (Lopes et al., 2009; Bassanezi et al., 2020), being transmitted from plant to plant by the Asian citrus psyllid *Diaphorina citri* Kuwayama (Hemiptera: Psyllidae) (Capoor et al., 1967).

The first visual symptoms of HLB, the typical leaf blotchy mottle or asymmetric chlorosis, usually appear on one or more branches several months after the plant is infected (Bové, 2006; Belasque Junior et al., 2009). After being passively introduced into the phloem tissue by *D. citri*, *C*Las persists in the shoot flushes until leaves mature enough to become a source (Alves et al., 2021a; Pandey et al., 2021). Then, it migrates from the scion to the root system, reaching titers high enough to cause damage to the roots even before symptoms become evident in the plant canopy (Johnson et al., 2014). From roots, *C*Las is translocated and irregularly colonizes the scion (Tatineni et al., 2008; Li et al., 2009), exhibiting an affinity for sink-developing tissues (Raiol-Junior et al., 2021). These characteristics of the pathosystem make the diagnosis of HLB difficult, and the adequate selection of tissue to be sampled for accurate *C*Las detection and to assess bacterial titer remains very important (Tatineni et al., 2008; Johnson et al., 2014; Raiol-Junior et al., 2021).

Blocking *C*Las movement to the roots is critical for disease progression, since HLB evolves into systemic deterioration of the citrus tree. The roots are a sink tissue that can be colonized early in the infection process, and there is further evidence that they act as a reservoir tissue for *C*Las replication and as a source of leaf infection (Johnson et al., 2014). The nonexistence of commercial citrus varieties and rootstocks resistant to *C*Las infection is one of the major limitations precluding a more sustainable HLB management. To overcome this situation, efforts have focused on identifying sources of *C*Las resistance within the germplasm of citrus-related species, intending to breed resistant varieties with commercial value (Ramadugu et al., 2016; Albrecht and Bowman, 2019). Among the resistant sources identified so far, some Oceanian citrus species and interspecific hybrids have attracted the attention of citrus breeders (Ramadugu et al., 2016; Alves et al., 2021b, 2022). These genotypes are particularly interesting because they are usually graft-compatible (Bitters, 2021; Siebert et al., 2015), and sexual hybridization is viable with other citrus types (Kalita et al., 2021). Therefore, they can be readily used in either genetic improvement programs or plant propagation, even though other horticultural traits must be observed, such as susceptibility to Citrus Tristeza virus (CTV), other citrus pests and diseases, abiotic stresses, and graft affinity (Smith et al., 2024).

Although the resistance of some of these Oceanian genotypes to *C*Las has been well documented in the canopy tissues (Alves et al., 2021b; 2022), many aspects regarding *C*Las infection and multiplication in the root system are unknown because they are either seedless or produce mainly monoembryonic seeds (Spiegel-Roy and Goldschmidt, 1996), and propagation through stem cuttings is usually difficult (Ahmed and Johnson, 2000; Hallaby, 2012). One method to propagate these genotypes in the short term, aiming to benefit from their *C*Las resistance, may be the interstocking of commercial citrus varieties, but the effectiveness of this propagation system to effectively block *C*Las movement within the grafted plant remains untested.

This work evaluated six HLB-resistant Oceanian genotypes as interstocks of HLB-susceptible ‘Valencia’ sweet orange scion, grafted onto HLB-susceptible ‘Rangpur’ lime rootstock under greenhouse conditions. *C*Las infection rate and titer were assessed over two years. Additionally, plant growth, root weight loss, biochemical and anatomical traits, and regulation of genes related to defense mechanisms that may be differentially expressed during the process of infection were investigated. To the best of our knowledge, this is the first report testing the use of truly HLB-resistant genotypes as citrus interstocks. Our findings revealed that some Oceanian interstock genotypes were able to significantly limit *C*Las movement from the infected scions to the roots of susceptible varieties. The potential benefits and limitations of this new approach to integrating HLB management and breeding are discussed.

## Materials and methods

### Local and plant maintenance

Experiments were carried out at the Fund for Citrus Protection (Fundecitrus) in the municipality of Araraquara, SP, Brazil (21°48’26” S, 48°09’54” W, 664 m.a.s.l.), since 2018 to 2023, in an insect-proof greenhouse with an average daily temperature varying between 17.9 °C minimum to 34.4 °C maximum, and 24.0 °C mean, and relative humidity varying between 41.5% minimum to 88.5% maximum, and 70.0% mean (**Supplementary F1**). Plants were grown in 5-L plastic bags filled with decomposed pine bark media and irrigated with a hose three times a week. The fertilization program was performed once a week with a nutrient solution containing 400 g of calcium nitrate, 600 g of magnesium sulfate, 144 g of potassium nitrate, 38 g of mono ammonium phosphate (MAP), 2.7 g of iron EDDHA, 3.12 g of copper EDTA, 0.72 g of zinc EDTA, 0.54 g of manganese EDTA, 2.1 g of boric acid, 0.36 g of sodium molybdate, and 40 mL per 1000 L of solution. The plants were sprayed with insecticides and miticides at intervals of 21 days as a preventive measure. The method employed for grafting was the inverted-T budding. All budwood was sourced from *C*Las-free mother trees cultivated in a greenhouse at Fundecitrus’ germplasm collection. One-year-old nucellar rootstocks were budded with interstock genotypes at 10–15 cm height above the root collar and 0.3-0.5 cm stem width. Rootstocks were looped to force bud growth. Whenever the interstock stem reached a length of approximately 35–40 cm and 0.3-0.5 cm thickness, the scion variety was budded. This interstock length was used to prevent any bacterial movement close to the bud union region as previously reported by Alves et al. (2021b). Interstocks were cut off just above the scion graft to force bud growth. Lateral sprouts were periodically removed by hand to train plants as single stems. Approximately 30 plants of each genotype were initially budded for propagation, considering a low budtake for most Oceanian genotypes.

### Plant material and experimental design

Since there was a wide variation in plant growth among the evaluated interstock genotypes, plants were budded at different moments, and two separate experiments were performed, grouping genotypes according to the budding date. In both experiments, ‘Valencia’ sweet orange IAC selection (*Citrus × aurantium* L. var. *sinensis* L.) was evaluated as the scion variety, and nucellar seedlings of the ‘Rangpur’ lime (*C.* × *limonia* Osbeck) were used as rootstocks. Both genotypes are well-known hosts of *C*Las, and ‘Valencia’ IAC is a commercial accession widely used in Brazil that is pre-immunized with a mild strain of CTV for cross-protection, as this is necessary for citrus cultivation in regions with CTV endemic conditions (Carvalho et al., 2019). Interstock treatments included some genotypes that were previously characterized as HLB-resistant by Alves et al. (2021b, 2022). In Experiment I, a *C. glauca* (Lindl.) Burkill × *Citrus* sp. hybrid (ADL-FDC12), *C. australasica* F. Muell. × [*C. australis* (A. Cunn. ex Mudie) Planch. × *C. australasica* F. Muell.] hybrid (AFL-BGC695), and *C. australis* (A. Cunn. ex Mudie) Planch. × *C. inodora* F.M. Bailey hybrid (ARL-LLA-FDC6) were evaluated as interstocks (**Table 1**). ‘Valencia’ IAC sweet orange (VSWO) was used as HLB-susceptible interstock control. In Experiment II, seven interstock genotypes were evaluated: an admixture hybrid of {*C. glauca* (Lindl.) Burkill × [*C. australis* (A. Cunn. ex Mudie) Planch. × *C. australasica* F. Muell.]} × *C. australasica* F. Muell. (ADL-BGC682), true-to-type *C. warburgiana* F.M. Bailey (NGL-FDC7), a *C. wintersii* Mabb. × *C. warburgiana* hybrid (BRFLxNWL-FDC2), AFL-BGC695, ARLxLLA-FDC6, ADL-FDC12 and VSWO (used as the control). In the case of Experiment II, interstocks showed a later and poorer plant growth; therefore, the budding height of the scion was variable among replications and shorter than 35 cm for some genotypes, because budding was adjusted to the interstock stem diameter available at the time of budding.

**Table 1:** List of genotypes that were evaluated as interstocks in experiments I and II, following the abbreviations as used at the Fundecitrus’ germplasm collection.

Common name	Abbreviation	Genotype
‘Valencia’ sweet orange	VSWO	*Citrus* × *aurantium* L. var. *sinensis* L. cv. ‘Valencia’ IAC
Australian desert lime hybrid	ADL-FDC12	*C. glauca* (Lindl.) Burkill × *Citrus* sp. hybrid
Australian desert lime hybrid	ADL-BGC682	{*C. glauca* × [*C. australis* (A. Cunn. ex Mudie) Planch. × *C. australasica* F. Muell.]} × *C. australasica* hybrid
Australian finger lime hybrid	AFL-BGC695	*C. australasica ×* (*C. australis × C. australasica*) hybrid
Australian round lime × Australian large-leaf wild lime	ARLxLLA-FDC6	*C. australis* × *C. inodora* F.M. Bailey hybrid
Brown River finger lime × New Guinea wild lime	BRFLxNWL-FDC2	*C. wintersii* Mabb. × *C. warburgiana* F.M. Bailey hybrid
New Guinea wild lime	NGL-FDC7	True-to-type *C. warburgiana*

**Figure 1:**
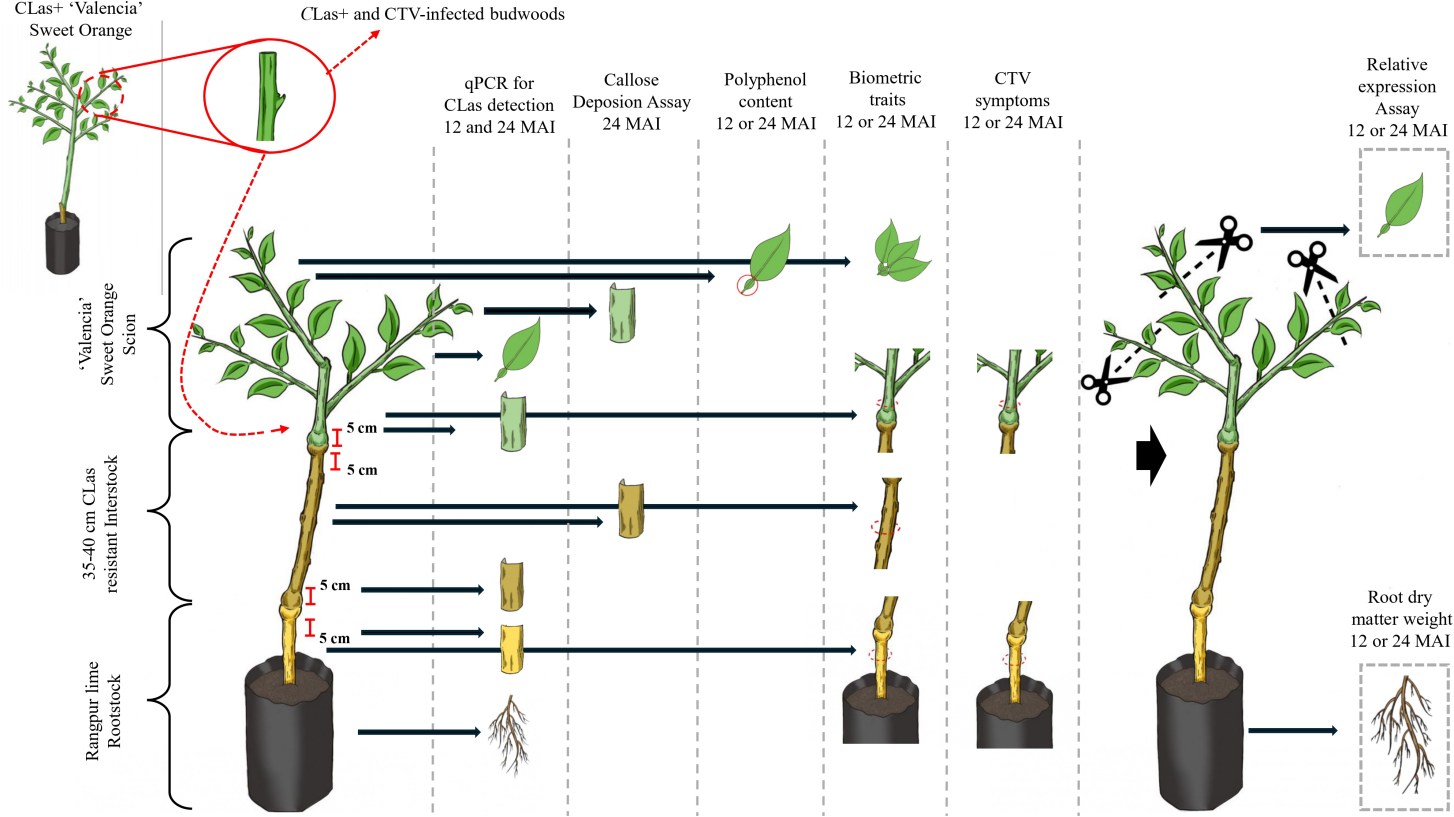
Schematic representation of the experimental workflow used to evaluate the influence of HLB-resistant interstock genotypes on *Candidatus* Liberibacter asiaticus (*C*Las) movement within citrus plants and other variables from 12 to 24 months after inoculation (MAI). An Oceanian genotype was placed as interstock between *C*Las-susceptible rootstock and scion varieties. Once grafts were taken, well-developed composite plants were inoculated with *C*Las-infected budwood in the scion by grafting.

In both experiments, the design was completely randomized with 4–25 replications of a single tree per *C*Las-challenge inoculated treatment. In our experimental scheme, the ‘Valencia’ sweet orange scion was inoculated with *C*Las-infected buds, and bacterial titer was assessed periodically from the scion to the roots, as well as the callose deposition and anatomical traits, total phenolic content of leaves, relative expression of selected target genes on the ‘Valencia’ scion, and weight of root dry matter. In addition, the plants were evaluated regarding biometric traits and CTV-induced stem pitting symptoms in the interstock and bud unions (**Figure 1**).

### Challenging the genotypes with *Candidatus* Liberibacter asiaticus Inoculation

When the scion showed mature (semi-hardwood) stem tissues and adequate plant growth (one and two years after scion budding for Experiments I and II, respectively), plants were challenge-inoculated with *C*Las. For this purpose, HLB-symptomatic ‘Valencia’ IAC sweet orange plants were kept in a greenhouse at Fundecitrus and used as an inoculum source. Budsticks used for inoculation were confirmed as positive for the presence of *C*Las, showing a mean qPCR cycle of quantification (Cq) of 23.0 ± 3.4 (Li et al., 2006). Two infected buds were grafted per tree following the methodology described by Lopes and Frare (2008). Graft inoculation was performed on the scion stem at approximately 5 cm above the bud union with the interstock genotype. The infected buds were kept throughout the experiment; however, they did not sprout and develop further since they consisted of buds with removed meristems, being used only as a source of *C*Las inoculation. These inoculum graft parts of the scion tissue were not sampled. Four to 25 trees were inoculated per treatment (except for ADL-FDC12 in experiment II, for which only two plants were inoculated). At least five trees per treatment remained as not inoculated to be evaluated as a healthy control.

### Sampling of plant tissues, qPCR for *C*Las detection and quantification

The movement of *C*Las within plant organs in various combinations of rootstock/interstock/scion was evaluated by monitoring the bacterial titer through qPCR analysis of DNA extracted from different tissues. In experiment I, plants were sampled at 12 and 24 months after *C*Las inoculation (MAI), and the following tissues were collected: 1-cm long apical segments of roots (new rootlets were prioritized and collected around the root ball); 1-cm long bark strips of the rootstock at 5 cm below the rootstock/interstock bud union; 1-cm long bark strips of the interstock at 5 cm above rootstock/interstock bud union; 1-cm long bark strips of the scion at 5 cm above the interstock/scion bud union; and 3–5 leaves per VSWO scion collected from newly-emerged mature flushes (**Figure 1**). In experiment II, the same tissues were sampled, but only at 12 MAI. Samples were prepared and subjected to genomic DNA extraction using the CTAB method (CTAB 2%; NaCl 1.4 M; PVP 10000 2%; EDTA 0.5 M, pH 8; Tris–HCl 1 M, pH 8) with 0.2% β-mercaptoethanol (Murray and Thompson, 1980), as described by Teixeira et al. (2005). DNA quantity and quality were analyzed at a spectrophotometer (NanoDrop 1000, Thermo Fisher Scientific, USA), and plant tissues from *C*Las-inoculated and control plants were individually analyzed for *C*Las presence and titer by qPCR. Each reaction was composed of 6 µL of the TaqMan qPCR Master Mix Kit (Ambion/Thermo Fisher Scientific, USA), and 0.35 mM of each specific initiator/probe that amplifies the β-subunit ribonucleotide reductase of *C*Las (RNR) (Zheng et al., 2016) and 1 µL of DNA from each sampled tissue (leaves, bark, and roots), with an average concentration of 100–300 ng/µL, was used in a final volume of 12 µL per reaction. The qPCR reactions were performed using a StepOne™ Real-Time PCR System (Applied Biosystems/Thermo Fisher Scientific), under the following thermal cycling conditions: 50 °C for 2 min, followed by 95 °C for 10 min, and then 45 cycles of 95 °C for 15 s and 58 °C for 45 s. Samples collected from *C*Las-free and *C*Las-infected sweet orange stock trees at Fundecitrus were used as negative and positive controls of the analysis, respectively, and non-template controls were used for each run. For all the evaluations of *C*Las titer, samples were considered *C*Las-positive when their qPCR quantification (Cq) cycle was lower than 34.0, following the same parameters described by Alves et al. (2021b) and Lopes et al. (2013) for *C*Las quantification in plant tissues. At the end of qPCR reactions, *C*Las quantification was performed based on the linear relationship between Cq values and the RNR log, according to the equation: y = −0.2921Cq + 11.62, where y represents the value expressed on a logarithmic scale. The standard curve equation was developed for the specific laboratory conditions under which the reactions were performed and is described by Martins et al. (2024).

### Relative gene expression assays via RT-qPCR

Relative expression analyses of putative plant defense gene homologs from citrus were performed at the end of both experiments to compare VSWO scion responses to different treatments (interstocks), for both control and *C*Las-infected plants. The VSWO scion was pruned to induce the sprouting of new vegetative flushes, and three to five flush shoots were randomly sampled per plant at V2 stage (Cifuentes-Arenas et al., 2018). At least three biological replicates were analyzed per treatment. The sampled flushes were put in 2.0 mL microtubes, immediately placed in liquid nitrogen, and stored in an ultra-freezer (-80 °C) until RNA extraction. Total RNA was extracted using Trizol reagent (Thermo Fisher Scientific). RNA quality and quantity were verified at a spectrophotometer (NanoDrop 1000, Thermo Fisher Scientific). Total RNA was treated with DNase I (Thermo Fisher), and the cDNA synthesis was carried out from 1 μg of RNA using the ‘RT Improm-II Reverse Transcriptase’ kit (Promega) according to the manufacturer’s instructions. Primers from citrus homologs from genes that have been previously reported as markers of plant defense in model plants were selected (Francis et al., 2009; Mafra et al., 2012; Mafra et al., 2013; Oliveira et al., 2019) (**Supplementary T1**). The RT-qPCR assays were performed in triplicate for each selected primer pair, using SYBR green reagent (Thermo Fisher Scientific), and the runs were performed on a StepOne™ Real-Time PCR System (Applied Biosystems/Thermo Fisher Scientific). Relative expression was calculated based on the 2^-ΔΔCt^ method as described by Livak and Schmittgen (2001), and data normalization was performed with the endogenous GAPC2 (glyceraldehyde-3-phosphate dehydrogenase) and ACT (Actin 2) (Mafra et al., 2012). Heat maps were generated using *C*Las-free control plants (VSWO interstock), which served as the standard reference for the experiments.

### Callose deposition and sieve morphometric analyses

To determine callose deposition due to *C*Las infection in the interstock genotypes, bark strips (4 × 6 mm) were sampled from the interstock stem at half distance from the scion and rootstock bud unions in experiment I (**Figure 1**). Other plant tissues were not investigated for this purpose. Subsequently, the interstock bark samples were placed in 1.5 mL microtubes containing 400 µL of a phosphate buffer (Na_2_HPO_4_ and NaH_2_PO_4_) (pH 7.2) and 2.5% glutaraldehyde. After 4 h (overnight) at 4 °C, the samples were rinsed 3 times with phosphate saline buffer (pH 7.2) for 10 min, then washed from 5 to 7 times with distilled water for 10 min in each washing step, followed by chlorophyll extraction with organic solvent (acetone) at the following dilutions: 30, 50, 70, 95, and 100%, remaining in each of the different dilutions for 15 min. In the end, the samples were transferred to new screw-cap 2.0 mL microtubes containing acetone 100% and kept at room temperature (± 25 °C) until subsequent analysis. Scanning Electronic Microscopy (SEM) was performed as described by Sivager et al. (2021). Briefly, fully dehydrated samples were dried to a critical point in a critical point dryer (CPD) using liquid CO_2_ as the transitional fluid. Dried specimens were mounted on aluminum stubs and sputter-coated with gold to enhance conductivity. Imaging was then performed using a FEI Quanta 250 scanning electron microscope operated at 20 kV. Four to five independent preparations of tissue sections were used for each genotype for the SEM analysis, being collected from different replicates. *C*Las-free and *C*Las-inoculated interstocks of AFL-BGC695, ADL-FDC12, RLxLLA-FDC6, and VSWO (HLB-susceptible control) were evaluated. Experiment II was not included in the callose deposition assessment.

Because composite plants using ADL-BGC682, ARLxLLA-FDC6, and NGL-FDC7 showed lower *C*Las detection in the roots, sieve morphometric traits of those genotypes were assessed by microscopy analysis in comparison to HLB-susceptible VSWO trees to investigate whether their sieve characteristics could relate to the difficulty for *C*Las movement within the interstock genotype. For this specific analysis, leaf petioles were sampled from the *C*Las-free mother plants from each genotype at Fundecitrus’ germplasm collection, and the same procedures aforementioned were used. Quantitative morphometric analyses were performed on SEM micrographs using ImageJ software (NIH, USA). The following anatomical parameters were measured: total petiole cross-sectional area, cortex area, xylem area, average cortex cell size, average phloem cell size, and average pore size. Measurements were conducted using calibrated scale bars to ensure spatial accuracy. Tissue areas were delineated manually using the polygon selection tool, while cortex and xylem regions were distinguished based on cell morphology and anatomical positioning. For cell and pore dimensions, three representative structures were measured per biological replicate, with three petioles per preparation, and their mean value was used to reduce within-sample variability. A total of six biological replicates (n = 6) were analyzed per genotype.

### Polyphenol content assessment

To investigate plant defense-related biochemical responses of VSWO scion to *C*Las infection on different interstock genotypes, the content of total polyphenols was measured in leaves from experiment II. For that, 3 to 5 leaves were randomly collected around the scion and were then placed in paper bags and lyophilized. Subsequently, 0.5 g per sample was placed in 2.0 mL microtubes containing metal spheres (8 mm wide) and disrupted using a TissueLyser II (Qiagen, Hilden, Germany), until the consistency of a fine powder. Phenolic compounds were extracted using 80% acetone and 100% methanol to optimize the extraction process. For each method, 50 mg of plant powder was mixed with 1.5 mL of the respective solvent in 1.5 mL microtubes. Samples were incubated at 48 °C for 105 min, followed by overnight incubation at room temperature. After vortexing, the samples were centrifuged at 8,000 rpm for 10 min, and the supernatants were transferred to pre-weighed 1.5 mL microtubes. The extracts were dried under a fume hood at room temperature for 72 h, and the tubes were reweighed to determine the extracted phenolic mass. Phenolic content was quantified using the Folin-Ciocalteu method with a commercial kit (Bioquochem, KB03006) following the manufacturer’s protocol. A standard curve was performed to determine the phenolic compound concentrations. Experiment I was not included in this assessment.

### Biometric assessments, and visual evaluation of graft compatibility and CTV-induced stem pitting

To gain more information on plant growth and HLB damage in trees using different interstocks, biometric traits were evaluated. For that, the stem diameter of each plant component (rootstock, interstock, and scion) was measured with a caliper at 5 cm from each bud union; the stem length (rootstock, interstock, and scion, the latter being considered from the bud union up to the apex of the longest shoot) was measured with a tape measure; the number of vegetative shoots per plant canopy was counted, and three shoots randomly selected per canopy were measured with a ruler. Evaluations were recorded at 24 MAI in experiment I and at 12 MAI in experiment II, in which shoots were not measured. For measurements, we considered only plants that were confirmed qPCR-positive for *C*Las detection (Cq ≤ 34.0), which were compared to healthy control plants within the same genotype. Following the same rationale, root loss caused by *C*Las infection was evaluated at the end of each experiment. The plants were removed from the substrate, their root systems were carefully extracted, washed with water and soap, dried in an oven at 60 °C for 72 h, subsequently weighed using a digital scale, and *C*Las-free and *C*Las-infected trees were compared within each interstock genotype. Finally, a visual assessment of graft compatibility and CTV-induced stem pitting symptoms was performed on stems at the bud union between scion/interstock and interstock/rootstock, after bark removal following 20 min in an autoclave, as described by Meissner et al. (2002).

### Statistical analyses

The data of *C*Las titer, biometric traits, root dry matter, and CTV symptom grades were subjected to analysis of variance (ANOVA). Average *C*Las titer was calculated using only replications that tested positive for each sample tissue and assessment period. When significant differences were found, the means were compared by Tukey’s test (comparisons between interstock genotypes) or the t-test (*C*Las-infected groups versus control trees within the same interstock) (p < 0.05). Infection rate was calculated as the ratio between replications that tested positive to total replications available, and only plants with a confirmed *C*Las-positive scion were considered for calculations of infection rates within further plant tissues (interstock bark, rootstock bark, and roots). The polyphenol content was evaluated by Duncan’s *post hoc* test followed by the Kruskal-Wallis’ test (p < 0.05). These statistical tests were performed using GraphPad Prism version 10.3.1 for Windows (GraphPad Software, Boston, Massachusetts, USA). Results from sieve morphometric traits are reported as mean ± standard deviation (SD), with statistical differences between genotypes being assessed by one-way ANOVA followed by Tukey’s *post hoc* test in R software, with significance set at p < 0.05.

## Results

### Detection and quantification of *Candidatus* Liberibacter asiaticus in different plant tissues

In experiment I, *C*Las was successfully graft-inoculated in the scion, with infection ranging from 58% (ARLxLLA-FDC6) to 92% (ADL-FDC12) of total plants per genotype at 12 and 24 MAI. In both assessment periods, *C*Las was not detected in any tissue of either plants with *C*Las-negative scions (failed inoculations) or control plants. In plants with *C*Las-positive scions, bacteria were detected in virtually all sampled tissues of all VSWO and ADL-FDC12 interstocked plants. A partial blockage of *C*Las translocation was observed in plants with an ARL-LLA-FDC6 interstock at 12 MAI, because bacteria were not detected in any interstock bark, but in 9% and 18% of rootstock barks and roots, respectively. For plants with an AFL-BGC695 interstock, *C*Las was detected in 66% of rootstock tissues. For the other interstock genotypes, *C*Las translocation was observed in all sampled root tissues. At 24 MAI, *C*Las detection rates were very similar or slightly higher than those observed at 12 MAI, indicating that this period is sufficient for the intended evaluation. At both assessment periods, *C*Las titers in the scion variety and rootstock bark were similar, independently of the interstock genotype used, except for lower titers with AFL-BGC695. In the interstock bark, VSWO and AFL-BGC695 showed the highest and lowest bacterial titers, respectively, and there was no detection of *C*Las in ARL-LLA-FDC6, at both 12 and 24 MAI. In the roots of the ‘Rangpur’ lime rootstock, regardless of the period and interstock genotype assessed, *C*Las titers did not differ among treatments evaluated and were generally high (**Table 2**).

**Table 2:** Proportion of detection (positive trees/total trees) and average titers (Log ± SE) of ‘*Candidatus* Liberibacter asiaticus’ (*C*Las) by qPCR analyses of different plant tissues (scion barks, scion leaves, interstock barks, rootstock barks, and roots) of different genotypes grafted onto ‘Rangpur’ lime rootstock, using ‘Valencia’ sweet orange as scion, at 12 and 24 months after *C*Las inoculation by grafting in experiment I.

Experiment I - 12 months after inoculation												
IG	Scion leaves			Interstock bark			Rootstock bark			Root		
	*C*Las+	%	Log ± SE	*C*Las+	%	Log ± SE	*C*Las+	%	Log ± SE	*C*Las+	%	Log ± SE
	Freq.			Freq.			Freq.			Freq.		
VSWO	12/20	60	7.06 ± 0.14 a	12/12	100	5.22 ± 0.08 a	12/12	100	4.69 ± 0.09 a	12/12	100	5.14 ± 0.22 a
ADL-FDC12	23/25	92	6.71 ± 0.12 ab	23/23	100	4.55 ± 0.08 b	23/23	100	4.94 ± 0.07 a	23/23	100	5.25 ± 0.16 a
AFL-BGC695	9/13	69	5.96 ± 0.58 b	6/9	66	4.12 ± 0.14 c	9/6	66	4.12 ± 0.14 b	6/9	66	4.72 ± 0.27 a
ARLxLLA-FDC6	11/19	58	6.40 ± 0.15 ab	0/11	0	nd	11/1	9	4.62 ab	2/11	18	5.72 ± 0.80 a
Experiment I - 24 months after inoculation												
IG	Scion bark			Interstock bark			Rootstock bark			Root		
	*C*Las+	%	Log ± SE	*C*Las+	%	Log ± SE	*C*Las+	%	Log ± SE	*C*Las+	%	Log ± SE
	Freq.			Freq.			Freq.			Freq.		
VSWO	12/20	60	6.51 ± 0.06 ab	12/12	100	6.07 ± 0.09 a	12/12	100	5.69 ± 0.15 a	11/12	92	4.53 ± 0.31 a
ADL-FDC12	22/24	92	6.53 ± 0.08 a	22/22	100	5.61 ± 0.09 b	22/22	100	5.72 ± 0.11 a	21/22	95	4.99 ± 0.26 a
AFL-BGC695	9/13	69	5.89 ± 0.38 b	5/9	55	5.05 ± 0.20 c	9/7	77	4.74 ± 0.33 b	6/9	66	4.49 ± 0.36 a
ARLxLLA-FDC6	11/19	58	6.10 ± 0.17 ab	0/11	0	nd	11/1	9	4.66 ab	2/11	18	5.59 ± 0.10 a

IG: interstock genotype; MAI: months after inoculation with *C*Las; VSWO: *Citrus ×aurantium* L. var. *sinensis* L. ‘Valencia’’ sweet orange; ADL-FDC12: *C. glauca* (LindI.) Burkill × *Citrus* sp hybrid; ARLxLLA-FDC6: *C. australis* (A. Cunn. ex Mudie) Planch. *× C. inodora* F.M. Bailey hybrid; AFL-BGC695: *C. australasica* F. Muell. × (*C. australis* (A. Cunn. ex Mudie) Planch. × *C. australasica* F. Muell.) hybrid *C*Las+: proportion of qPCR-positive trees; Nd = undetected. ± = standard error of the mean. Means followed by the same lowercase letters in columns do not differ by the Tukey’s test (p ≤ 0.05).

In experiment II, at 12 MAI, the lowest *C*Las infection rate of the scion variety was observed for plants with NGL-FDC7 interstock, with only 58% of scions testing positive for *C*Las by qPCR. In contrast, 70% of scions with an ADL-BGC682 interstock were infected, whereas other interstock genotypes showed more than 90% of trees with infected scions. None of the control plants showed *C*Las in their tissues. Plant tissues were completely colonized by *C*Las in most genotypes evaluated, except in plants with an ADL-BGC682 interstock, which showed the lowest detection rates among all the interstock and rootstock tissues at 12 MAI. In this interstock genotype, only 14% of plants presented *C*Las in their roots, which corresponded to a single tree. Compared to experiment I, average infection rates increased for plants with an ARLxLLA-FDC6 interstock, even though 42% of plants were still not infected with *C*Las in their root tissues. Other Oceanian interstocks evaluated varied from 73% (NGL-FDC7) to 82% (AFL-BGC695) of root infection, or none of the roots/rootstock barks remained free of *C*Las (ADL-FDC12 and BRFLxNWL-FDC2), which were all within the infection range of the VSWO control. Bacterial titers did not present relevant differences among treatments evaluated across all tissues sampled, including the *C*Las-susceptible scion and rootstock tissues, and titers were the highest in the scion tissues. Interestingly, *C*Las detection and titers were lowest in the bark tissues of the interstock ADL-BGC682 when compared with the other genotypes (**Table 3**). Individual datasets for each replicate plant can be assessed at **Supplementary T2_IE1 and IE2**.

**Table 3:** Proportion of detection (positive trees/total trees) and average titers (Log ± SE) of ‘*Candidatus* Liberibacter asiaticus’ (*C*Las) by qPCR analyses in plant tissues (scion leaves, interstock barks, rootstock barks, and roots) of different genotypes grafted onto ‘Rangpur’ lime, rootstock, using ‘Valencia’ sweet orange as scion, at 12 months after *C*Las inoculation by grafting in experiment II.

IG	Scion leaves			Interstock bark			Rootstock bark			Root		
	*C*Las+	%	Log ± SE	*C*Las+	%	Log ± SE	*C*Las+	%	Log ± SE	*C*Las+	%	Log ± SE
	Freq.			Freq.			Freq.			Freq.		
VSWO	9/9	100	6.71 ± 0.20 a	9/9	100	5.26 ± 0.17 a	9/9	100	4.58 ± 0.25 b	7/9	78	4.95 ± 0.42 a
ADL-FDC12	2/2	100	6.35 ± 0.29 a	2/2	100	4.66 ± 0.52 a	2/2	100	3.85 ± 0.10 b	2/2	100	3.94 ± 1.00 a
ADL-BGC682	7/10	70	7.04 ± 0.17 a	2/7	29	4.44 ± 0.28 a	1/7	14	4.99 ab	1/7	14	5.46 a
AFL-BGC695	11/11	100	7.32 ± 0.15 a	11/11	100	4.73 ± 0.33 a	10/11	91	4.99 ± 0.11 ab	9/11	82	5.76 ± 0.07 a
ARLxLLA-FDC6	12/13	92	7.13 ± 0.09 a	08/12	67	5.02 ± 0.39 a	5/12	42	5.00 ± 0.36 ab	7/12	58	4.51 ± 0.52 a
BRFLxNWL-FDC2	4/4	100	7.03 ± 0.11 a	4/4	100	5.70 ± 0.31 a	4/4	100	5.77 ± 0.15 a	4/4	100	5.97 ± 0.08 a
NGL-FDC7	11/19	58	6.44 ± 0.50 a	10/11	91	4.86 ± 0.28 a	8/11	73	4.80 ± 0.27 ab	8/11	73	4.89 ± 0.40 a

IG: interstock genotype; MAI: months after inoculation with *C*Las; VSWO: *Citrus ×aurantium* L. var. *sinensis* L. ‘Valencia’ sweet orange; ADL-FDC12: *C. glauca* (LindI.) Burkill × *Citrus* sp hybrid; ARLxLLA-FDC6: *C. australis* (A. Cunn. ex Mudie) Planch. *× C. inodora* F.M. Bailey hybrid; AFL-BGC695: *C. australasica* F. Muell. × (*C. australis* (A. Cunn. ex Mudie) Planch. × *C. australasica* F. Muell.) hybrid ADL-BGC682: [*C. glauca* (Lindl.) Burkill × (*C. australis* (A. Cunn. ex Mudie) Planch. × *C. australasica* F.Muell.)] × *C. australasica* F.Muell. hybrid; BRFLxNWL-FDC2: *C. wintersii* Mabb. × *C. warburgiana* F.M. Bailey. Hybrid; NGL-FDC7*: C. warburgiana* F.M. Bailey. *C*Las+: proportion of qPCR-positive trees; Nd = undetected. ± = standard error of the mean. Means followed by the same lowercase letters in columns do not differ by the Tukey’s test (p ≤ 0.05).

### Relative gene expression assays

**Figure 2:**
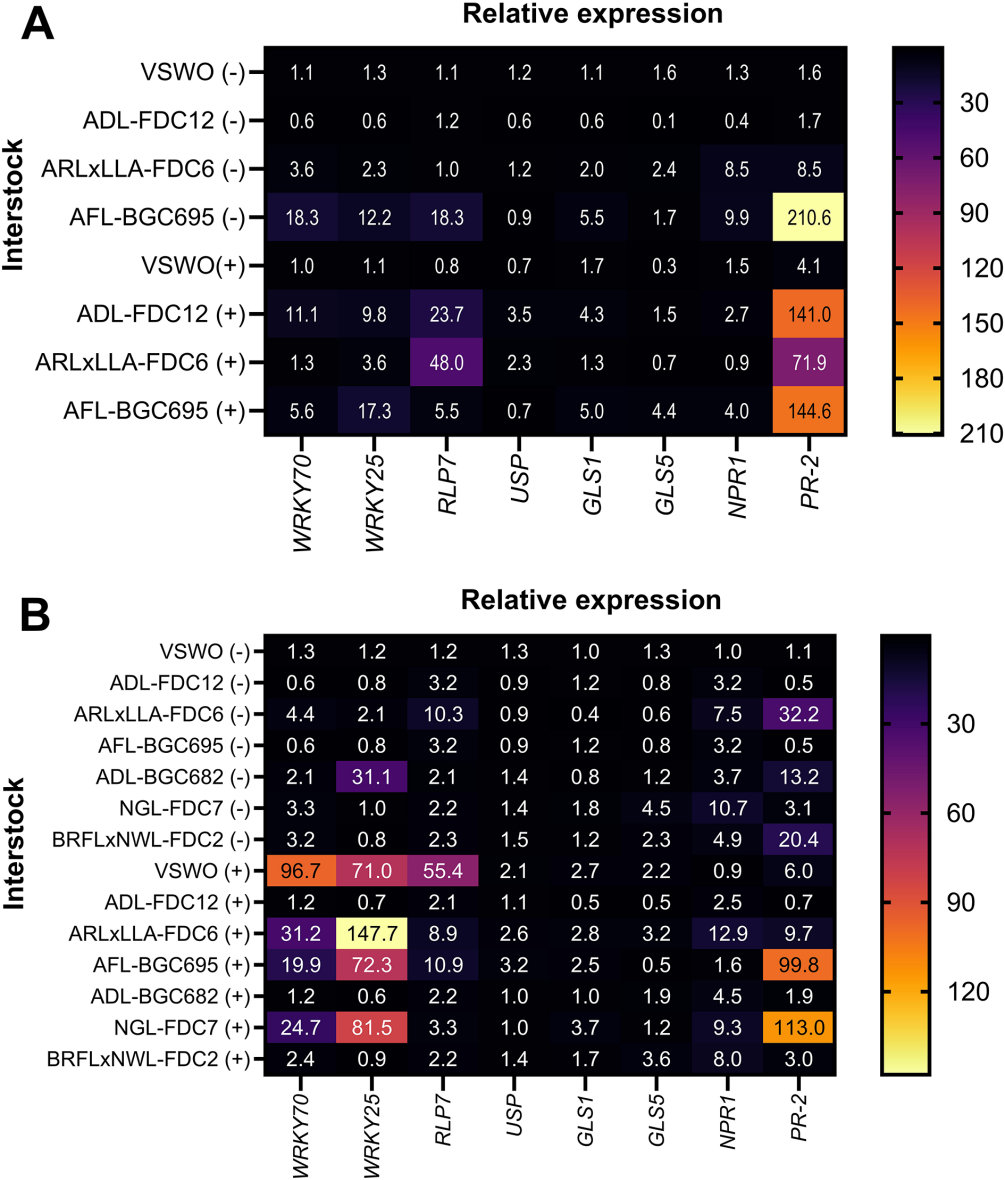
Relative expression of citrus homologs of genes related to plant defense in leaves of ‘Valencia’ sweet orange scion grafted onto ‘Rangpur’ lime rootstock using different genotypes at the interstock position after infection with *Candidatus* Liberibacter asiaticus (*C*Las) in experiment I at 24 months after inoculation (MAI) **(A)** and in experiment II at 12 MAI **(B)**. (-): *C*Las-free (control) trees; (+): *C*Las-positive trees. Genotype details in **Table 1**.

In experiment I, the relative expression of eight defense-related gene homologs was assessed at 24 MAI, comparing all treatments (infected or not with *C*Las) to VSWO (-) control plants as the standard. The *Pathogenesis-Related Protein-2* (*PR-2*) stood out among the evaluated targets, with 4.14 ± 0.80; 90 ± 8.61; and 140.97 ± 8.79 log-fold change for *C*Las-infected VSWO, ARLxLLA-FDC6, and ADL-FDC12 interstock genotypes, respectively (**Figure 2a**). *PR-2* was always upregulated in plants with an AFL-BGC695 interstock, for which the relative expression reached 210.59 ± 6.60 in *C*Las-negative plants and 144.63 ± 8.61 in *C*Las-infected plants. For the other evaluated target gene homologs, variations in expression levels were observed, but at much lower levels, either due to the presence of the *C*Las or due to the different genomic background of the interstock genotypes compared to VSWO (-) control plants (**Figure 2a**).

In experiment II, relative gene expression was assessed at 12 MAI, and up-regulation regarding target genes was observed. In general, mostly in *C*Las-infected plants and mainly those comprising VSWO, AFL-BGC682, BRFLxNWL-FDC2, and NGL-FDC7 interstocks, there were higher relative expression values for the target gene homologs of *WRKY70*, *WRKY25*, *RPL7*, and *PR-2* in relation to *C*Las-free VSWO (-) control. For the other evaluated target genes, the overall expression level changes were much lower (**Figure 2b**). Although *C*Las-free plants with an Oceanian interstock usually presented up-regulation of defense genes compared to VSWO (-) control plants, most of the increase in relative expression occurred after *C*Las challenge-inoculation (**Figure 2b**).

### Callose deposition and sieve morphometric analyses

**Figure 3:**
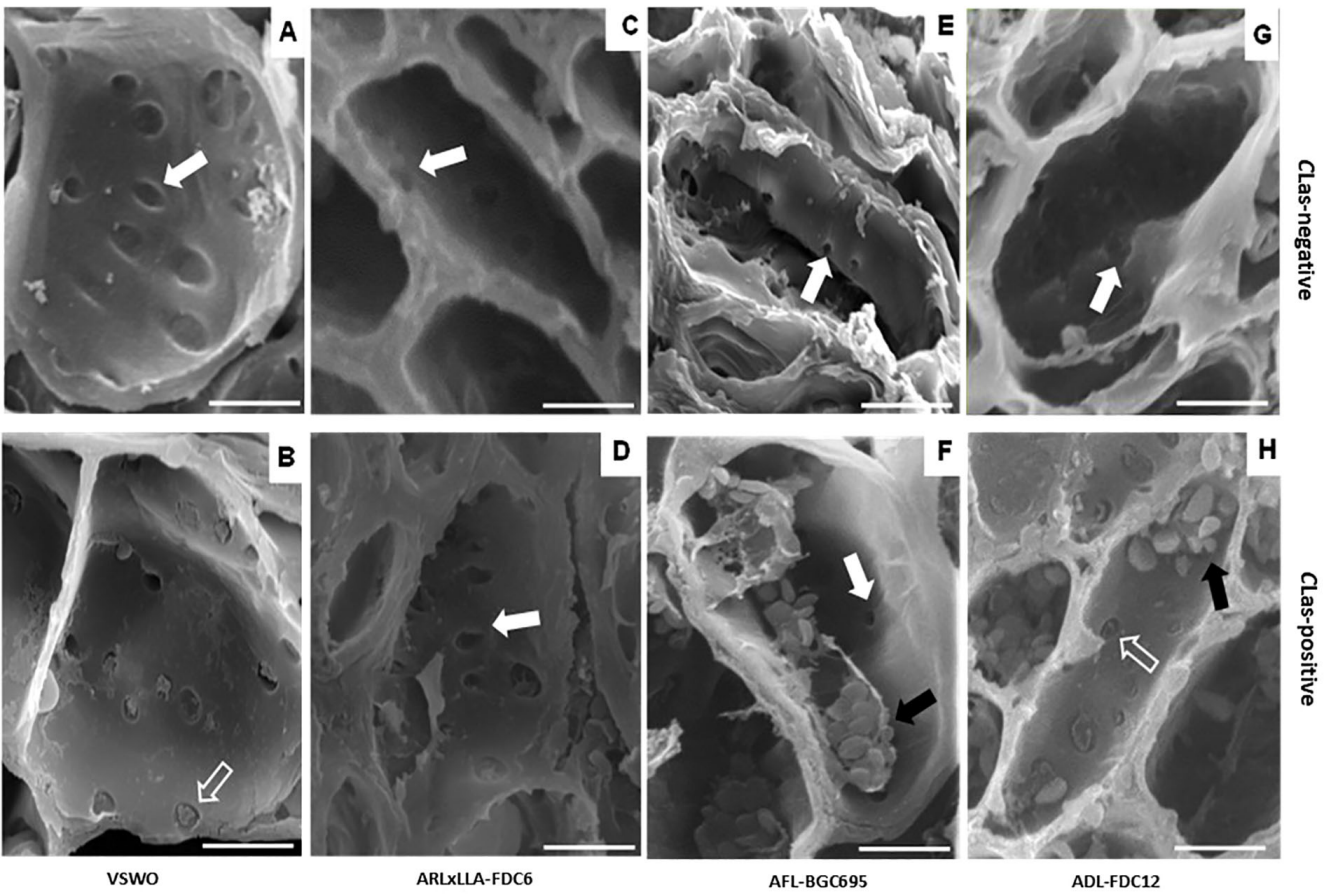
Scanning Electron Microscope (SEM) images of phloem cells in the bark of interstock genotypes VSWO **(A, B)**, ARLxLLA-FDC6 **(C, D)**, AFL-BGC695 **(E, F)**, and ADL-FDC12 **(G, H)**, intergrafted onto ‘Rangpur’ lime with ‘Valencia’ sweet orange as the scion, 24 months after CLas inoculation. Scions in panels **(A, C, E, G)** are *C*Las-negative; those in **(B, D, F, H)** are *C*Las-positive. White arrows indicate unblocked phloem pores; open arrows indicate callose deposition **(D)**; black arrows indicate starch grains. Genotype details in **Table 1**.

The phloem cells of interstock barks were analyzed to observe the effects of *C*Las infection on the callose deposition and anatomical changes in experiment I (**Figure 3**). In control plants, there was neither callose nor plugging of the pores at the sieve plate of all interstock genotypes evaluated, and there was also no accumulation of starch (**Figures 3A, C, E, G**). On the other hand, only the control interstock (VSWO) and ADL-FDC12 showed callose or plugging of the pores in the presence of *C*Las (**Figures 3B, H**, respectively), which were the same treatments with the highest bacterial titer and movement within plant tissues (**Table 2**). Despite the presence of *C*Las in the scion and rootstock tissues, no anatomical damage nor plugging of the pores was observed in the phloem cells of ARLxLLA-FDC6 and AFL-BGC695 interstock barks (**Figures 3D, F**, respectively). The accumulation of starch grains was observed in only two genotypes, AFL-BGC695 and ADL-FDC12 (**Figures 3F, H**, respectively).

Analysis of the pore surface area/cell surface area ratio in phloem cells from *C*Las-free and *C*Las-infected plants was also performed in samples from interstock barks of VSWO, ARLxLLA-FDC6, AFL-BGC695, and ADL-FDC12 in experiment I (**Table 4**). In most accessions, ratios decreased in *C*Las-infected compared to *C*Las-free plants due to an increase in the phloem cell area in the infected samples, except for ARLxLLA-FDC6. Remarkably, the ratio for VSWO presented the largest relative decrease upon *C*Las infection, highlighting the impact of the bacterium on this highly susceptible genotype. Oceanian genotypes had significantly lower ratios than VSWO in *C*Las-free plants, but infected plants did not differ.

**Table 4:** Ratio of pore surface area to cell surface area in phloem cells of the interstock bark in control (*C*Las-) and *C*Las-infected (*C*Las+) using different genotypes grafted onto ‘Rangpur’ lime, using ‘Valencia’ sweet orange as scion, 24 months after grafting inoculation with ‘*Candidatus* Liberibacter asiaticus’.

IG	*C*Las -	*C*Las +
VSWO	0.03911 ± 0.01182b	0.01984 ± 0.00862a
ARLxLLA-FDC6	0.01132 ± 0.00298a	0.01890 ± 0.0069a
AFL-BGC695	0.015001 ± 0.00256a	0.01498 ± 0.00389a
ADL-FDC12	0.01894 ± 0.00688a	0.01217 ± 0.00643a

IG: interstock genotype; MAI: months after inoculation with *C*Las; VSWO: *Citrus ×aurantium* L. var. *sinensis* L. ‘Valencia’’ sweet orange; ADL-FDC12: *C. glauca* (LindI.) Burkill × *Citrus* sp hybrid; ARLxLLA-FDC6: *C. australis* (A. Cunn. ex Mudie) Planch. *× C. inodora* F.M. Bailey hybrid; AFL-BGC695: *C. australasica* F. Muell. × (*C. australis* (A. Cunn. ex Mudie) Planch. × *C. australasica* F. Muell.) hybrid.; Values represent the mean ratio followed by the standard error of the mean (±). They were calculated based on the mean pore measurements from five different cells. Means followed by the same lowercase letters in columns do not differ by Tukey’s test (p ≤ 0.05).

After the evaluation of the infection rates for different plant tissues in both experiments (**Tables 2** and **3**), ADL-BGC682, ARLxLLA-FDC6, and NGL-FDC7 were selected as the most promising interstock genotypes due to their lower *C*Las infection rates in the roots. The sieve morphometric traits in leaf petioles of these genotypes were compared to those of sweet orange as a *C*Las-susceptible control (**Table 5**). Samples were collected from *C*Las-free mother plants of all genotypes to characterize the typical traits of each genotype without the influence of the HLB disease. Interestingly, Oceanian genotypes presented in general phloem pore size and other sieve traits with considerably smaller dimensions than the VSWO control, which reinforces that this trait may influence the flux of bacterial cells along the phloem, making their movement more difficult.

**Table 5:** Sieve morphometric traits in leaf petioles of *C*Las-free plants of Oceanian citrus hybrids in comparison to ‘Valencia’ sweet orange trees.

IG	Petiole area (mm²)	Cortex area (mm²)	Xylem area (mm²)	Phloem area (mm²)	Cortex cell size (µm²)	Xylem cell size (µm²)	Phloem cell size (µm²)	Pore size (µm²)
ADL-BGC682	0.383 ± 0.055c	0.005 ± 0.002b	0.077 ± 0.021b	0.05 ± 0.014b	93.523 ± 38.256bb	88.234 ± 10.681b	518.411 ± 153.105bb	1.926 ± 0.416b
ARLxLLA-FDC6	0.914 ± 0.315b	0.008 ± 0.002b	0.075 ± 0.02b	0.056 ± 0.02b	72.521 ± 14.195b	92.026 ± 26.959b	247.9 ± 76.775b	1.515 ± 0.200b
NGL-FDC7	0.896±0.28b	0.016±0.007b	0.122±0.049bb	0.081±0.032bb	135.464±57.042b	130.774±35.69bb	596.978± 169.685b	1.929 ± 0.280b
VSWO	1.325 ± 0.106b	0.025 ± 0.012b	0.171 ± 0.048b	0.116 ± 0.022b	77.244 ± 26.275b	177.083 ± 54.129b	576.71 ± 257.050b	2.692 ± 0.552b

IG: interstock genotype; VSWO: *Citrus* × *aurantium* L. var. sinensis L. ‘Valencia’’ sweet orange; ADL-BGC682: [*C. glauca* (Lindl.) Burkill × (*C. australis* (A. Cunn. ex Mudie) Planch. × *C. australasica* F. Muell.)] × *C. australasica* F. Muell. hybrid; ARLxLLA-FDC6: *C. australis* (A. Cunn. ex Mudie) Planch. *× C. inodora* F.M. Bailey hybrid; NGL-FDC7*: C. warburgiana* F.M. Bailey. ± = standard error of the mean. Means followed by the same lowercase letters in columns do not differ by Tukey’s test (p ≤ 0.05).

### Polyphenol content assessment

**Figure 4:**
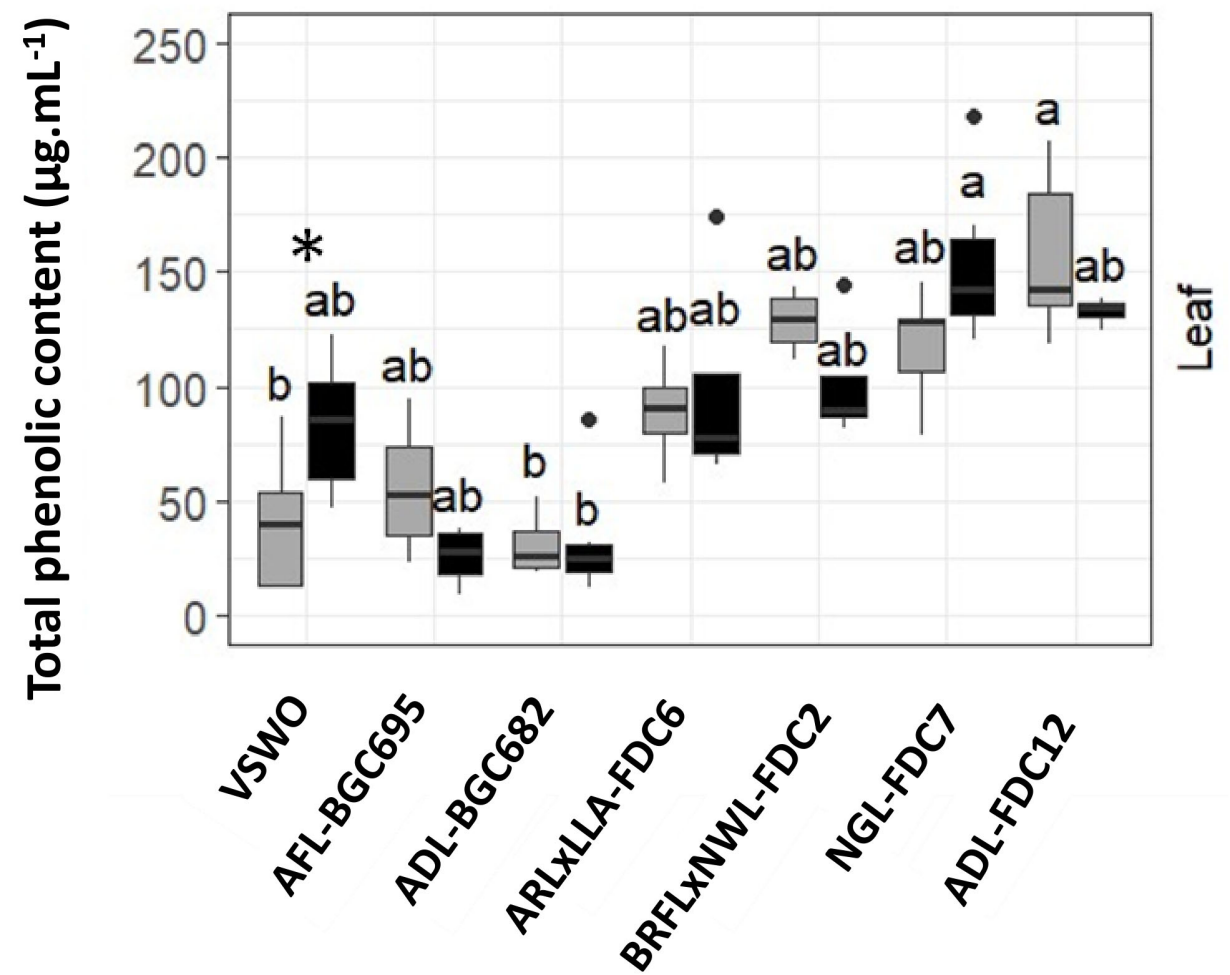
Total phenolic content (µg·mL^-^¹) in leaf tissues of control (gray boxes) and *C*Las-inoculated plants (black boxes) across interstock combinations. Boxplots show polyphenol distribution: boxes = interquartile range, horizontal line = median, whiskers = 1.5× IQR, dots = outliers. Different letters indicate significant differences between interstocks (p < 0.01; Kruskal-Wallis + Duncan). * denotes significant differences between control and *C*Las-infected VSWO leaves within each interstock (p < 0.05). Genotype details in **Table 1**.

Total polyphenol content analyses were carried out in leaves of both *C*Las-infected and control VSWO plants in experiment 2 to investigate further plant defense responses induced by the different interstock genotypes. Total polyphenol contents were only increased after *C*Las infection in the leaves of plants with a VSWO interstock (HLB-susceptible control). In other interstock genotypes, polyphenol contents were not significantly different between *C*Las-infected and control plants, even though average contents were generally higher than in VSWO, regardless of *C*Las infection, except for AFL-BGC695 and ADL-BGC682 interstocks, which presented the lowest average contents of total polyphenols (**Figure 4**).

### Biometric assessments, and visual evaluation of graft compatibility and CTV-induced stem pitting

**Figure 5:**
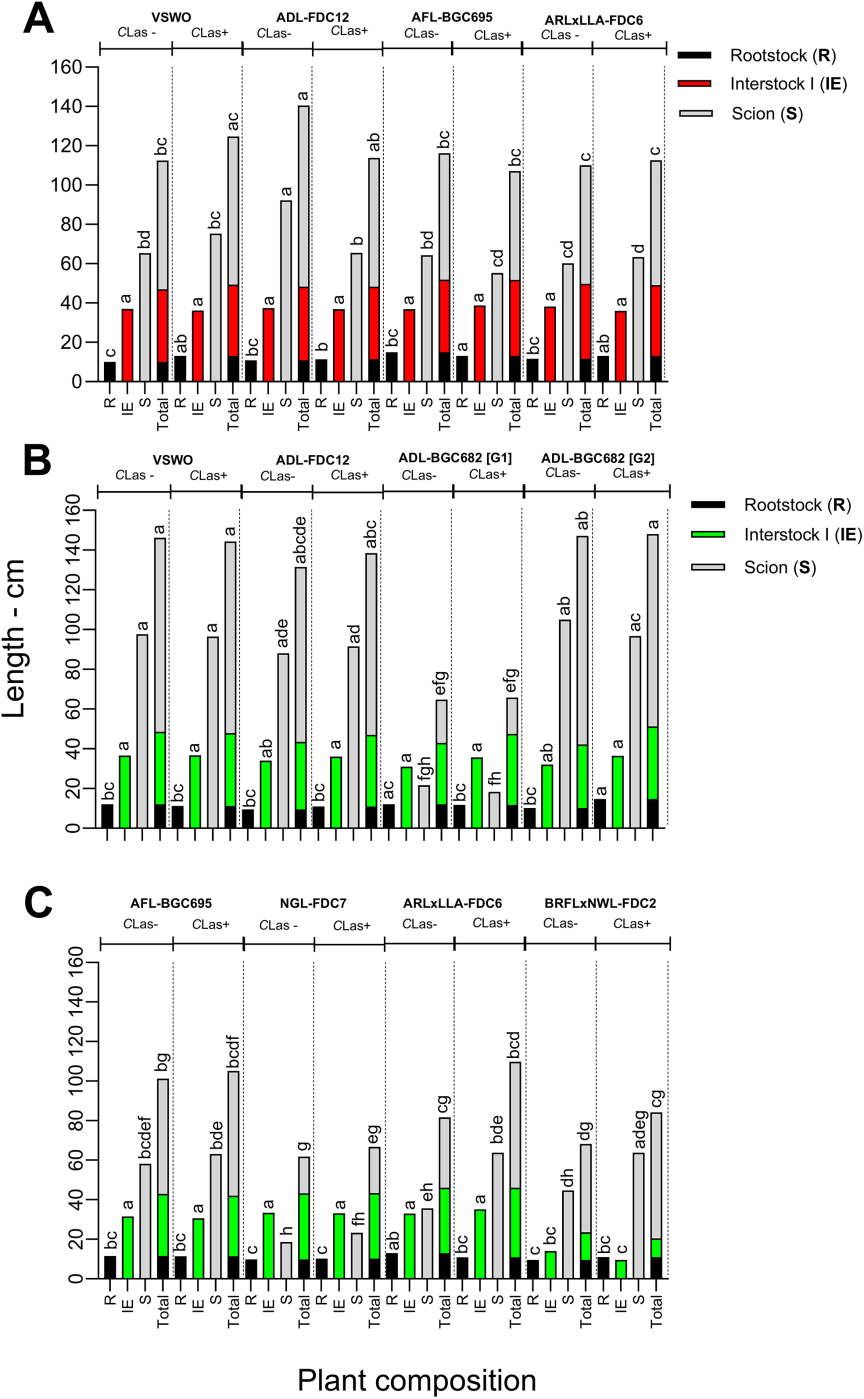
Rootstock, interstock, and scion length (cm) of ‘Valencia’ sweet orange trees grafted onto ‘Rangpur’ lime rootstock using different genotypes at the interstock position in non-infected (-) and *Candidatus* Liberibacter asiaticus (*C*Las)-infected (+) plants in experiments I (**A**, 24 months after inoculation) and II (**B, C**, 12 months after inoculation). *C*Las+: proportion of qPCR-positive trees. Means followed by the same lowercase letters in different plant portions (rootstock, interstock, scion, and total) do not differ by Tukey’s test (p ≤ 0.05). G1: group 1 of vigor. G2: group 2 of vigor. Genotype details in **Table 1**.

Plant growth was assessed for three plant components (rootstock, interstock, and scion) for a better understanding of the effect of the treatments on biometric variables. In experiment I, *C*Las-infected plants exhibited a higher rootstock size in comparison to non-infected plants (F = 8.066, df= 71, p < 0.0001, **Figure 5a**), due to the selection of slightly more developed individuals at the inoculation time to facilitate budding of the *C*Las-inoculum source. In terms of the scion length, *C*Las-free ADL-FDC12 had the largest canopy (F = 9.652, df= 71, p < 0.0001). Overall, total plant height was similar among all evaluated genotypes (**Supplementary F2**). In experiment II, ADL-BGC682, plants had more variable sizes and were divided into two groups of vigor to allow a better comparison (**Figure 5b**). *C*Las-infected ADL-BGC682 plants of group 2 [G2] and *C*Las-free ARL-LLA-FDC6 had the largest rootstocks, whereas *C*Las-free NGL-FDC7 and BRFLxNWL-FDC2 treatments had the smallest ones (**Figure 5c**). Once again, these differences in rootstock size arose mainly from the plant selection at the time of inoculation. The interstock length of all BRFLxNWL-FDC2 plants was shorter than that of other genotypes evaluated. *C*Las inoculation did not significantly affect the scion length, and treatments could be grouped as follows: the most vigorous genotypes included VSWO, ADL-FDC12, and ADL-BGC682 [G2]; medium-sized genotypes were ARL-LLA-FDC6, BRFLxNWL-FDC2, and AFL-BGC695; and genotypes with a very low vigor consisted of NGL-FDC7 and ADL-BGC682 [G1].

Overall, stem diameter decreased from the rootstock to the scion, but it was uniform among the evaluated genotypes regardless of *C*Las infection. In experiment I, the ‘diameter index’ (DI) of the different combinations assessed ranged from 0.78 to 1.14 for rootstock/interstock and interstock/scion bud unions, respectively, indicating a normal range for graft compatibility between the evaluated genotypes. This was supported by the visual analysis of the bud unions, which did not present typical symptoms of incompatibility; it is important to point out that our results indicate no short−term graft incompatibility, though the potential influence of CTV on graft union morphology cannot be determined (**Supplementary F3**). Newly-emerged shoots on the scion canopy were also counted and measured to evaluate the impact on plant growth upon *C*Las infection. The shoot length was shorter in the *C*Las-infected plants compared to the control ones within the same interstock genotype in all treatments. *C*Las-infected plants of VSWO and ADL-FDC12 produced more vegetative shoots per tree than healthy plants. For AFL-BGC695 and ARLxLLA-FDC6, there were no significant differences between healthy and challenge-inoculated plants at 24 MAI. In experiment II, the DI was also similar among treatments and ranged from 0.59 to 1.067, except for NGL-FCD7, which had the lowest means. In this trial, the length of shoots was not measured. Concerning the number of shoots that sprouted on the scion canopy, none of the treatments statistically differed at 12 MAI, except for the *C*Las-infected ADL-BGC682 [G1] (**Table 6**).

**Table 6:** Stem diameter of rootstock, interstock, and scion, diameter index, length, and number of shoots of different genotypes grafted onto ‘Rangpur’ lime, using ‘Valencia’ sweet orange as scion, in ‘*Candidatus* Liberibacter asiaticus’ (*C*Las)-free and *C*Las-positive plants.

Experiment I - 24 months after inoculation (MAI)									
IG	*C*Las	Diameter (mm)						Shoot length (cm)	Shoot number
		RS	IS (+5)	IS (-5)	SC	DI RS/IS	DI IS/SC		
VSWO	(-)	13.62 ± 0.54 ab	10.65 ± 0.55 bcd	8.76 ± 0.73 cd	8.22 ± 0.61 ab	0.78 ± 0.04 c	0.94 ± 0.02 ab	15.73 ± 0.88 aA	8 ± 1.58 cB
VSWO	(+)	13.99 ± 0.32 a	12.48 ± 0.42 ab	9.59 ± 0.22 bc	9.12 ± 0.12 ab	0.9 ± 0.04 bc	0.96 ± 0.02 ab	10.28 ± 0.66 cB	15.25 ± 1.18 bA
ADL-FDC12	(-)	12.97 ± 0.57 ab	14.15 ± 0.46 a	11.24 ± 0.38 ab	9.96 ± 0.26 a	1.1 ± 0.02 a	0.89 ± 0.01 b	16.67 ± 1.16 aA	10.71 ± 1.49 bcB
ADL-FDC12	(+)	13.74 ± 0.26 a	13.52 ± 0.32 a	11.15 ± 0.22 a	9.5 ± 0.42 a	0.98 ± 0.01 ab	0.85 ± 0.04 b	9.03 ± 0.36 cB	20.64 ± 1.01 aA
AFL-BGC695	(-)	13.1 ± 0.43 ab	12.2 ± 0.67 ab	10.9 ± 1.00 abc	9.12 ± 0.43 ab	0.93 ± 0.03 bc	0.85 ± 0.06 b	14.27 ± 0.57 abA	10.8 ± 1.74 bcA
AFL-BGC695	(+)	12.58 ± 0.53 ab	11.28 ± 0.65 bc	9.23 ± 0.45 c	8.08 ± 0.27 ab	0.9 ± 0.03 bc	0.88 ± 0.02 b	9.04 ± 1.13 cB	8.56 ± 0.85 cA
ARL-LLA-FDC6	(-)	11.41 ± 0.15 bc	9.1 ± 0.63 cd	6.8 ± 0.30 d	7.72 ± 0.34 ab	0.8 ± 0.05 c	1.14 ± 0.06 a	10.83 ± 0.92 bcA	4.8 ± 0.86 cA
ARL-LLA-FDC6	(+)	10.58 ± 0.18 c	8.87 ± 0.27 d	6.86 ± 0.32 d	7.86 ± 0.26 b	0.84 ± 0.03 c	1.11 ± 0.06 a	7.79 ± 0.80 cB	6.27 ± 0.59 cA
Experiment II - 12 months after inoculation (MAI)									
IG	*C*Las	Diameter (mm)						Shoot length (cm)	Shoot number
		RS	IS (+5)	IS (-5)	SC	DI RS/IS	DI IS/SC		
VSWO	(-)	16.03 ± 0.32 a	13.51 ± 0.32 a	10.73 ± 0.11 ab	10.16 ± 0.16 a	0.84 ± 0.02 abcd	0.95 ± 0.02 b	-	4.00 ± 0.55 abA
VSWO	(+)	14.60 ± 0.35 a	12.62 ± 0.42 a	10.30 ± 0.33 b	9.67 ± 0.30 a	0.86 ± 0.02 abcd	0.94 ± 0.01 b	-	3.78 ± 0.40 abA
ADL-FDC12	(-)	13.27 abcd	13.01 ab	11.76 abc	10.22 ab	0.98 abcdef	0.87 ab	-	4.00 ab A
ADL-FDC12	(+)	12.70 ± 1.66 abcd	12.86 ± 1.23 a	10.27 ± 1.16 abc	8.32 ± 0.64 abc	1.02 ± 0.04 ab	0.81 ± 0.03 ab	-	5.00 ± 2.00 abA
ADL-BGC682 [G1]	(-)	7.13 ± 0.81 f	4.17 ± 0.35 d	3.97 ± 0.68 e	5.89 ± 1.56 bdce	0.60 ± 0.10 def	1.67 ± 0.70 a	-	1.67 ± 0.67 abB
ADL-BGC682 [G1]	(+)	6.32 ± 0.96 f	5.04 ± 0.56 de	4.26 ± 0.42 ef	4.52 ± 0.56 cde	0.81 ± 0.04 abcdef	1.08 ± 0.18 ab	-	4.67 ± 2.31 abA
ADL-BGC682 [G2]	(-)	13.42 ± 0.26 abc	14.58 ± 0.67 a	13.10 ± 0.41 ab	11.22 ± 1.47 a	1.09 ± 0.07 abc	0.86 ± 0.14 ab	-	6.00 ± 1.00 aA
ADL-BGC682 [G2]	(+)	13.87 ± 0.31 ab	14.71 ± 0.18 a	12.98 ± 0.68 a	10.38 ± 0.33 a	1.06 ± 0.02 a	0.80 ± 0.02 b	-	3.75 ± 0.38 abA
AFL-BGC695	(-)	10.93 ± 0.18 bcde	8.88 ± 0.69 bc	7.23 ± 0.64 cdef	5.72 ± 0.19 bcde	0.81 ± 0.06 abcde	0.81 ± 0.09 ab	-	2.00 ± 0.41 abA
AFL-BGC695	(+)	10.10 ± 0.47 bcde	9.05 ± 0.51 bc	7.63 ± 0.47 cd	5.94 ± 0.47 bcde	0.89 ± 0.02 ab	0.78 ± 0.03 b	-	2.36 ± 0.24 abA
ARL-LLA-FDC6	(-)	9.03 ± 0.46 df	6.73 ± 0.91 cd	5.55 ± 0.53 de	5.67 ± 0.80 bcde	0.75 ± 0.10 bcdef	1.01 ± 0.05 ab	–	2.33 ± 0.33 abA
ARL-LLA-FDC6	(+)	11.28 ± 0.54 bcd	8.63 ± 0.36 bc	6.90 ± 0.34 def	7.38 ± 0.42 bc	0.78 ± 0.04 bcde	1.08 ± 0.05 ab	–	2.83 ± 0.27 abA
BRFLxNWL-FDC2	(-)	9.79 ± 0.20 bcdef	6.62 ± 1.59 cd	6.74 ± 1.86 cde	5.28 ± 0.94 bcde	0.68 ± 0.18 bcdef	0.81 ± 0.08 ab	–	2.50 ± 0.50 abA
BRFLxNWL-FDC2	(+)	10.64 ± 0.46 bcde	7.84 ± 0.45 cde	7.65 ± 0.40 cd	7.21 ± 0.90 abcd	0.74 ± 0.01 bcdef	0.95 ± 0.12 ab	–	3.00 ± 0.41 abA
NGL-FCD7	(-)	8.24 ± 0.53 ef	4.87 ± 0.43 d	4.40 ± 0.35 e	4.39 ± 0.56 de	0.59 ± 0.02 ef	0.99 ± 0.07 ab	–	1.60 ± 0.24 bA
NGL-FCD7	(+)	8.62 ± 0.19 ef	5.07 ± 0.26 d	4.64 ± 0.30 e	4.39 ± 0.28 e	0.59 ± 0.02 ef	0.97 ± 0.06 ab	–	1.91 ± 0.21 bA

IG: interstock genotype; (-): *C*Las-negative ‘Valencia’ sweet orange trees; (+): *C*Las-positive ‘Valencia’ sweet orange trees; RS: rootstock; IS: interstock; SC: scion; (+5): average IS diameter at 5 cm above the RS/IS junction; (-5): average IS diameter at 5 cm below the IS/SC junction; DI: diameter index, the ratio between the RS and IS diameters at (+5) or the ratio between the SC and IS diameters at (-5); ± = standard error of the mean; VSWO: *Citrus ×aurantium* L. var. *sinensis* L. ‘Valencia’ sweet orange; ADL-FDC12: *C. glauca* (LindI.) Burkill × *Citrus* sp hybrid; ARLxLLA-FDC6: *C. australis* (A. Cunn. ex Mudie) Planch. *× C. inodora* F.M. Bailey hybrid; AFL-BGC695: *C. australasica* F. Muell. × (*C. australis* (A. Cunn. ex Mudie) Planch. × *C. australasica* F. Muell.) hybrid ADL-BGC682: [*C. glauca* (Lindl.) Burkill × (*C. australis* (A. Cunn. ex Mudie) Planch. × *C. australasica* F. Muell.)] × *C. australasica* F. Muell. hybrid; BRFLxNWL-FDC2: *C. wintersii* Mabb. × *C. warburgiana* F.M. Bailey hybrid; NGL-FDC7*: C. warburgiana* F.M. Bailey. Different uppercase letters indicate a difference between healthy and *C*Las-positive plants within the same IG by the t-test (p ≤ 0.05). Different lowercase letters indicate differences between the evaluated genotypes by the Tukey test (p ≤ 0.05). (-) not evaluated.

For all the evaluated combinations at the end of experiment I (24 MAI), the root dry matter (RDM) was lower in *C*Las-infected plants compared to the respective and non-infected interstock genotype. Regardless of the *C*Las infection, plants with VSWO and ADL-FDC12 interstocks had higher RDM than ARLxLLA-FDC6 and AFL-BGC695 (**Table 7**; **Supplementary F2**). For the ARLxLLA-FDC6 interstock genotype, it was possible to analyze data from *C*Las-infected trees that showed infection only in the scion variety, and plants with bacteria in all plant organs. In this case, regardless of the bacterium detection in the scion only or in both the scion and roots, there was a loss of around 60% of RDM in the root system compared to control trees. In experiment II, only *C*Las-infected VSWO showed a significant decrease in RDM at 12 MAI compared to the control counterpart. For the other interstocks evaluated, RDM alterations were not consistent between *C*Las-positive and corresponding control plants within the same interstock. Overall, RDM was higher in VSWO and ADL-FDC12 than in the other Oceanian genotypes, and notably, when compared with NGL-FDC7 and ADL-BGC682 [G1], validating the previous plant growth evaluations (**Figure 4**; **Table 6**). For *C*Las-infected ARLxLLA-FDC6, the RDM was similar between plants with either the *C*Las-detection in only the scion or both scion and roots (**Table 7**).

**Table 7:** Root dry matter (RDM) of different genotypes grafted onto ‘Rangpur’ lime, using ‘Valencia’ sweet orange as scion, in ‘*Candidatus* Liberibacter asiaticus’ (*C*Las)-free and *C*Las-positive plants.

Experiment I - 24 Months after inoculation (MAI)			
IG	*C*Las (-)	*C*Las (+)	RDM loss (%)
VSWO	50.32 ± 7.02 aA	23.43 ± 4.16 aB	53
ADL-FDC12	42.80 ± 8.08 aA	21.47 ± 1.58 abB	50
ARLxLLA-FDC6	29.53 ± 1.19 aA	13.36 ± 0.01 abB^1^	55
		10.77 ± 2.48 abB^2^	64
AFL-BGC695	34.54 ± 5.84 aA	10.91 ± 0.82 bB	68
Experiment II - 12 Months after inoculation (MAI)			
IG	*C*Las (-)	*C*Las (+)	RDM loss (%)
VSWO	97.46 ± 13.41 aA	61.15 ± 11.64 aB	37
ADL-FDC12	19.31 bA	16.64 ± 3.58 bcA	14
ARLxLLA-FDC6	17.53 ± 2.25 bA	24.09 ± 3.02 bcA ^1^	nd
		22.59 ± 2.87 bcA^2^	nd
AFL-BGC695	24.36 ± 5.70 bA	19.79 ± 2.68 bcA	19
ADL-BGC682 [G1]	7.85 ± 0.75 bA	4.29 ± 5.75 bcA	45
ADL-BGC682 [G2]	43.63 ± 9.42 bA	44.61 ± 5.14 abA	nd
BRFLxNWL-FDC2	24.94 ± 11.45 bA	23.56 ± 5.80 bcA	5
NGL-FDC7	5.50 ± 0.74 bA	6.84 ± 1.44 cA	nd

IG: interstock genotype; MAI: months after *C*Las graft inoculation. VSWO: *Citrus ×aurantium* L. var. *sinensis* L. ‘Valencia’ sweet orange; ADL-FDC12: *C. glauca* (LindI.) Burkill × *Citrus* sp hybrid; ARLxLLA-FDC6: *C. australis* (A. Cunn. ex Mudie) Planch. *× C. inodora* F.M. Bailey hybrid; AFL-BGC695: *C. australasica* F. Muell. × (*C. australis* (A. Cunn. ex Mudie) Planch. × *C. australasica* F. Muell.) hybrid ADL-BGC682: [*C. glauca* (Lindl.) Burkill × (*C. australis* (A. Cunn. ex Mudie) Planch. × *C. australasica* F. Muell.)] × *C. australasica* F. Muell. hybrid; BRFLxNWL-FDC2: *C. wintersii* Mabb. × *C. warburgiana* F.M. Bailey hybrid; NGL-FDC7*: C. warburgiana* F.M. Bailey. G1: ADL-BGC682 plants in the low vigor group; G2: ADL-BGC682 plants in the high vigor group (-): *C*Las-negative ‘Valencia’ sweet orange trees; (+): *C*Las-positive ‘Valencia’ sweet orange trees; ± = standard error of the mean. Nd: a net root loss was not detected. ^1^ Means of plants with both *C*Las-positive scion and roots; ^2^ Means of plants with *C*Las-positive scion but *C*Las-negative roots. Different uppercase letters in rows indicate different inoculation means within interstock genotypes by the t-test (p ≤ 0.05) and for ARLxLLA-FDC6 treatments by the Tukey’s test (p ≤ 0.05). Different lowercase letters in columns indicate statistically significant differences in interstock means within control and *C*Las-inoculated treatments, as determined by Tukey’s test (p ≤ 0.05).

It is important to note that, regardless of *C*Las infection, there was a poor growth of the root system when most Oceanian genotypes were used as interstocks (**Supplementary F2**). Since all budwood propagated in Brazil is pre-immunized with a mild CTV strain for cross-protection, we evaluated CTV symptoms on the Oceanian citrus interstocks. Indeed, more severe CTV-induced stem pitting symptoms were observed in most Oceanian citrus interstock stems (**Table 8**, **Supplementary F3**). However, the severity of CTV-induced stem pitting was not affected by *C*Las-infection regardless of the interstock genotype used. Moreover, graft incompatibility symptoms were not observed at the bud union of any plant (**Supplementary F3**).

**Table 8:** Visual assessment of CTV-induced stem pitting on stems at the bud union of scion/interstock and interstock/rootstock of different genotypes grafted onto ‘Rangpur’ lime, using ‘Valencia’ sweet orange as scion, in non-infected and ‘*Candidatus* Liberibacter asiaticus’ (*C*Las)-infected plants.

Experiment I - 24 months after inoculation		
Interstock	*C*Las (-)	*C*Las (+)
VSWO	1 ± 0.00b	1.13 ± 0.14b
ADL-FDC12	1.17 ± 0.17b	1.38 ± 0.19b
ARLxLLA-FDC6	4 ± 0.50a	4.63 ± 0.18a
AFL-BGC695	4.33 ± 0.31a	4.08 ± 0.33a
Experiment II - 12 months after inoculation		
Interstock	*C*Las (-)	*C*Las (+)
VSWO	1.13 ± 0.18c	1.17 ± 0.14c
ADL-FDC12	2.5 ± 0.29bc	2.75 ± 0.18b
ARLxLLA-FDC6	4.5 ± 0.71ab	4.69 ± 0.21a
AFL-BGC695	4.25 ± 0.29ab	4.13 ± 0.08a
ADL-BGC682 [G1]	3 ± 0.47ab	3.88 ± 0.22ab
ADL-BGC682 [G2]	4.88 ± 0.10a	4.75 ± 0.18a
BRFLxNWL-FDC2	2.75 ± 0.61bc	4.5 ± 0.30a
NGL-FDC7	3 ± 0.58ab	4.13 ± 0.40a

VSWO: *Citrus ×aurantium* L. var. *sinensis* L. ‘Valencia’ sweet orange; ADL-FDC12: *C. glauca* (LindI.) Burkill × *Citrus* sp hybrid; ARLxLLA-FDC6: *C. australis* (A. Cunn. ex Mudie) Planch. *× C. inodora* F.M. Bailey hybrid; AFL-BGC695: *C. australasica* F. Muell. × (*C. australis* (A. Cunn. ex Mudie) Planch. × *C. australasica* F. Muell.) hybrid ADL-BGC682: [*C. glauca* (Lindl.) Burkill × (*C. australis* (A. Cunn. ex Mudie) Planch. × *C. australasica* F. Muell.)] × *C. australasica* F. Muell. hybrid; BRFLxNWL-FDC2: *C. wintersii* Mabb. × *C. warburgiana* F.M. Bailey hybrid; NGL-FDC7*: C. warburgiana* F.M. Bailey. G1: ADL-BGC682 plants in the low vigor group; G2: ADL-BGC682 plants in the high vigor group (-): *C*Las-negative ‘Valencia’ sweet orange trees; (+): *C*Las-positive ‘Valencia’ sweet orange trees; ± = standard error of the mean. Means followed by the same letter in columns do not differ significantly from each other according to Tukey’s test (p ≤ 0.05).

## Discussion

Clear phenotyping for *C*Las resistance and susceptibility, as well as consequent genetic improvement of Aurantioideae for breeding either tolerance (presence of bacteria in the genotype without causing economic losses or relevant symptoms) or resistance (lack of survival and multiplication of bacteria in the genotype), are priority strategies for the sustainable management of HLB in the long term. Such characterization is instrumental to be applied in both conventional and biotechnological breeding approaches. Recent studies have indicated varying levels of resistance to *C*Las within Oceanian citrus types and some interspecific hybrids between them and Asian citrus types, compared to current commercial varieties of citrus (Ramadugu et al., 2016; Albrecht and Bowman, 2019; Alves et al., 2021b, 2022; Mahmoud and Dutt, 2025; Zhao et al., 2025; Younas et al., 2025). Resistance of specific Oceanian genotypes was proven by challenge-inoculating *C*Las into a susceptible ‘Rangpur’ lime rootstock and evaluating the colonization of several genotypes at the scion position, including susceptible sweet orange scions used as controls (Alves et al., 2021b). Full resistance was identified in some Oceanian genotypes that did not show *C*Las multiplication in the scion, except for eventual detection of *C*Las close to the bud union, 5–15 cm above the highly infected ‘Rangpur’ lime rootstock. Aiming to evaluate whether these promising HLB-resistant Oceanian genotypes would block *C*Las movement from HLB-susceptible ‘Valencia’ sweet orange scion into ‘Rangpur’ lime rootstock when a resistant interstock was used, we designed an experimental setup under controlled greenhouse conditions. The interstocks exhibited an average length of approximately 35 cm across all evaluated combinations to avoid eventual translocation of bacterial cells close to the bud union, except for BRFLxNWL-FDC2, in which the mean interstock length was less than 20 cm due to poor plant growth. With this approach, a successful blockage by the interstock could lead to the propagation of readily available commercial citrus varieties with a long-lasting, uninfected root system.

Our results demonstrate that none of the evaluated Oceanian genotypes tested were able to completely block the movement of *C*Las from the scion of ‘Valencia’ sweet orange into the rootstock tissues of ‘Rangpur’ lime, up to 12–24 months after the scion inoculation by grafting infected budwood. However, some genotypes, namely ADL-BGC682 and ARLxLLA-FDC6, were able to considerably limit infection of the roots because 42 to 86% of trees with an infected VSWO scion did not show *C*Las multiplication in the HLB-susceptible rootstock below the HLB-resistant interstocks. In a previous study, we also observed that no *C*Las was detected in the root system of ‘Valencia’ sweet orange grafted onto ADL-BGC682 evaluated as rootstock (Darolt et al., 2025), which reinforces this hybrid’s high level of HLB resistance (Alves et al., 2021b, 2022). The two selected genotypes consist mainly of an admixture of *Citrus australis*, *C. inodora*, and *C. glauca*. Other genotypes involving mainly *C. wintersii*, *C. warburgiana*, *C. australasica*, and even a F1 hybrid of *C. glauca* with *Citrus* sp. showed much higher levels of bacterial movement from the VSWO scion to the rootstock, in addition to *C*Las titers similar to those of trees with a *C. sinensis* interstock (VSWO, control treatment). In some individuals, the presence of *C*Las was detected only in interstock or rootstock bark tissues but not in the rootlets. However, even a few plants of VSWO interstock tested negative for *C*Las in the roots, which could be explained by the irregular distribution of the bacterium in infected tissues.

The restriction of *C*Las movement appears to be related to sieve pore size, based on morphometric data from leaf petioles of the interstocks evaluated in Experiment I. The hybrids, particularly ARLxLLA-FDC6, exhibited smaller structural dimensions in the vascular tissues, including reduced phloem area, cortex cell size, phloem cell size, xylem cell size, and pore size. Smaller pores can mechanically restrict the passage of *C*Las, thereby reducing its mobility between tissues; however, the available studies correlate this mechanical restriction with callose deposition rather than with anatomical characteristics themselves (Koh et al., 2012; Achor et al., 2020). Moreover, ARLxLLA-FDC6 showed an absence of callose deposition. In contrast, the control interstock (VSWO) and ADL-FDC12 displayed callose accumulation or pore plugging in the presence of *C*Las, and these were the same treatments associated with the highest bacterial titers and greater pathogen movement within plant tissues. It is well established that the presence of *C*Las triggers structural reorganization of citrus phloem cells, including callose deposition. According to Achor et al. (2020), who evaluated microscopy images from flushes, bark, mature-leaf midribs, and roots, this represents the main plugging mechanism in HLB-infected flushes, where it reduces the open space of the pores. The authors also observed that deposition varied dramatically among the different tissues examined. Based on our data, it is possible to infer that pore size was more directly related to the blockage of *C*Las movement. Since increased callose synthesis and deposition are a general defense response to plant pathogens (Ellinger and Voigt, 2014), the absence of callose deposition in ARLxLLA-FDC6 may indicate a tolerant mechanism. Clearly, detailed microscopy analyses of calloses deposition in additional genotypes used as interstocks would help support this hypothesis, such as the case of ADL-BGC682, which was not evaluated in the present study. In addition, it would be necessary to repeat trials over longer periods, both under greenhouse and field conditions, integrating this strategy with other established approaches such as vector control of *D. citri*. Furthermore, the same genotypes and other promising ones should be evaluated in combination with HLB-resistant or tolerant scions, to observe the interaction of all these associated factors with the plant’s response to *C*Las infection and movement.”.

In the context of HLB resistance, *C*Las titers were usually higher in ‘Valencia’ sweet orange scion tissues than in the Oceanian interstock tissues, but titers across *C*Las-infected ‘Valencia’ sweet orange scions and ‘Rangpur’ lime rootstocks were similar regardless of the interstock used. Moreover, trees with a *C*Las-infected scion had smaller root weight at 24 MAI regardless of the infection status of the rootstock in experiment I. Altogether, these findings suggest that the level of HLB resistance of the Oceanian interstock genotype does not significantly influence the susceptibility of either the scion or the rootstock of citrus varieties in greenhouse conditions. Furthermore, there was no evidence of translocation of antimicrobial and antiproteolysis peptides from the *C*Las-resistant interstock tissues to ameliorate disease defense either in the scion or in the rootstock tissue, despite recent evidence that such molecules are related to HLB resistance in some Australian limes (Younas et al., 2025). However, since tree age and size at infection moment relates to HLB severity and influence *C*Las lateral movement within trees (Bové, 2006; Raiol-Junior et al., 2021), and the root system seems to play a major role in bacterial redistribution within infected trees (Johnson et al., 2014), only long-term evaluation under field conditions can confirm whether HLB progress and severity may be significantly influenced by the use of effectively resistant interstocks in comparison to conventional graft combinations. The detrimental effects of the *C*Las infection on root weight were more evident in experiment I than in experiment II, most likely due to the longer time elapsed between inoculation and the final assessment of the plants in the former experiment. Specifically, to ensure that the use of resistant interstocks is effective in limiting *C*Las movement, we evaluated Experiment I between 12 and 24 MAI. Previous studies have shown that *C*Las infection has a detrimental impact on the root system (Cavichioli et al., 2024), and these effects are known to be time−dependent, becoming more evident after prolonged periods of infection (Pulici et al., 2022). In fact, the lack of consistent RDM difference in Experiment II (12 MAI) is likely due to the shorter assessment time compared to Experiment I (24 MAI). In line with this, our results corroborate the hypothesis that root damage caused by *C*Las accumulates over time, reinforcing the importance of long−term evaluations to fully understand the impact of the disease. Nevertheless, no drastic changes were observed for either the number of *C*Las-positive plants or bacterial titers between the two evaluation times. For this reason, experiment II was ended 12 MAI.

Regarding the interaction between CTV and *C*Las and their association with root system damage or influence on plant growth, visual assessment of CTV-induced stem pitting at the bud union of scion/interstock and interstock/rootstock showed that, except for ADL-BGC682 and ARLxLLA-FDC6, the other evaluated interstocks exhibited high severity of CTV-induced stem pitting compared to VSWO, independent of *C*Las infection. Concerning the possible link between CTV-induced stem pitting and RDM loss, in Experiment I, the effect of *C*Las infection was clear, independent of CTV infection. In Experiment II, however, except for the control, the other evaluated genotypes did not show reduced RDM when comparing *C*Las-infected versus healthy ones, regardless of the level of CTV-induced stem pitting. As discussed previously, this is mainly associated with the fact that RDM loss is time-dependent, and the damage caused by *C*Las was more evident in Experiment I, which was conducted over a longer period. Interestingly, when analyzing total length, the genotypes ADL-BGC682 (G1) and NGL-FDC7 were the ones that exhibited poor growth. These results may be associated with trade-off parameters, such as *C*Las blockage in ADL-BGC682 occurring at the cost of increased CTV damage, which may also explain the poor growth observed in these genotypes, ADL-BGC682 (G1) and NGL-FDC7. Even so, these were not the groups that presented, statistically, the highest levels of induced stem pitting. Indeed, in this work, most Oceanian types presented similar severe stem pitting but varied CLas blockage. Therefore, experiments designed to evaluate the effects of CTV and *C*Las independently would provide a clearer understanding of the specific damage caused by each pathogen, allowing a more precise assessment of their individual contributions to root system impairment and growth reduction. Moreover, given the very specific reaction of each citrus genotype to each of these diseases, such damage needs to be necessarily interpreted individually for each evaluated citrus type and not extrapolated as a general response.

It is possible to infer, based on experimental evidence, that *C*Las blockage efficiency and RDM loss were not necessarily related. For the most promising interstocks, ARLxLLA-FDC6 and ADL-BGC682, it was observed that ARLxLLA-FDC6 exhibited 82% *C*Las blockage in root samples in Experiment I and an RDM reduction of 55% when compared to healthy plants within the group of *C*Las-infected plants. In Experiment II, the observed blockage decreased to 42% on *C*Las-infected plants compared to healthy ones, and there was no statistically significant difference in RDM between healthy and diseased plants. Meanwhile, ADL-BGC682, evaluated only in Experiment II, demonstrated 82% blockage, with no difference in RDM loss between healthy plants and those infected with *C*Las. These findings corroborate that root damage and loss caused by *C*Las accumulates over time, and based on our data, the efficiency of *C*Las blockage is not directly correlated with the observed RDM loss.

Notwithstanding, the fact that two Oceanian genotypes were able to block *C*Las infection of the rootstock partially opens the opportunity to test promising interstock combinations accordingly. Moreover, most biometric variables were not considerably affected by *C*Las infection in the greenhouse, even though infected plants produced more shoots per canopy that were generally shorter compared to the shoots of healthy trees. This growth pattern is similar to that observed in *C*Las-infected citrus trees in the field and greenhouse (Bové, 2006; Shahzad et al., 2023; Neupane et al., 2023). Cifuentes-Arenas et al. (2022) also observed on diseased trees that shoots increased by 20% on lemon and by 70% on sweet orange trees. Regarding *C*Las movement within infected plants, Alves et al. (2021b) reported a consistent *C*Las detection in the same Oceanian genotypes 5 cm above the graft union with a *C*Las-infected rootstock, and partial detection up to 15 cm of the scion height, but never in the upper leaves. The authors correlated this localized detection of *C*Las with the pressure of the phloem sap flow upwards from the rootstock to the scion. This motivated us to evaluate 35-cm-long interstocks, attempting to avoid the short movement of *C*Las. However, the detection of *C*Las in most rootstocks indicated that bacterial cells were able to translocate from the scion to the rootstock through the interstock. In other citrus pathosystems like tristeza and citrus sudden death, the use of resistant interstocks (5 to 15-cm long) was inefficient in blocking pathogens translocation into susceptible rootstocks and, thus, controlling these diseases (Pompeu Junior and Blumer, 2008; Folimonova et al., 2008). Bani Hashemian et al. (2010) found that *C. glauca* and *C. australis* were resistant to six citrus viroids, but long-distance movement and multiplication of pathogens were also detected in trees using both species as interstocks. The authors suggested that both species were poor hosts, but some viroid multiplication could occur, which is similar to our observations with *C*Las. On the other hand, because *C*Las movement was partially blocked by a 35-cm-long interstock in some Oceanian genotypes, alternative propagation methods could optimize *C*Las blockage by the most promising Oceanian interstocks, for instance, by top-working of large trees, which is a technique widely used in some citrus regions (Navarro, 2015). This remains to be evaluated.

Besides the reduction of the *C*Las infection, a composite plant using Asian and Oceanian stocks must present an overall viable tree performance for commercial cultivation. It is noteworthy to mention that plant growth was significantly influenced by the interstock genotype, as previously observed in field conditions (Martínez-Cuenca et al., 2016). Trees using the ‘Valencia’ sweet orange interstock had the highest plant growth, and only ADL-FDC12 (F1 hybrid of *C. glauca* with *Citrus* sp.) showed similar results, whereas other Oceanian genotypes induced poor growth in general. This was noticeable for *C. warburgiana*-based genotypes, as the interstock length was reduced after the first grafting on ‘Rangpur’ lime rootstocks and, later, the ‘Valencia’ sweet orange scion growth on the interstock was also poor, even making it difficult to perform *C*Las inoculation by grafting. Typical graft incompatibility symptoms were not observed; therefore, this poor growth may be genetic or more likely a result of the high susceptibility of Oceanian citrus to CTV (Spiegel-Roy and Goldschmidt, 1996). Most genotypes evaluated in this work showed severe pitting of the interstocks, indicating that their reaction to the CTV strain used for cross-protection as part of the citrus budwood propagation system of sweet orange in Brazil was too aggressive for most Australian limes. CTV replicates in the phloem companion cells and is transmitted either by grafting or naturally by aphids (Roistacher and Bar-Joseph, 1987); thus, the prevalence of CTV is a conditioning factor for the citrus industry in most citrus regions worldwide (Folimonova et al., 2020). Our results corroborate that only Oceanian CTV-tolerant or resistant hybrids that also conjugate good horticultural performance should be considered for HLB resistance improvement, as discussed by Smith et al. (2024). Otherwise, their cultivation must be performed only in areas free of CTV. Interestingly, the reaction to CTV was independent of the HLB resistance, because Oceanian genotypes with the most severe stem pitting presented less *C*Las in the roots, while ADL-FDC12 presented the least pitting but full infection by *C*Las. This suggests that CTV-induced stem pitting may be blocking *C*Las flux through the phloem, as pitting seriously compromises the size and structure of vascular tissue (Schneider, 1957; Khalilzadeh et al., 2024; Aldrich et al., 2024). ADL-FDC12 is an F1 hybrid between *Citrus* sp. and *C. glauca*, apparently tolerant to CTV, and VSWO scions on this interstock additionally presented the largest plant growth. Overall, our results suggest that broadening hybridization between Asian citrus types and Australian limes may be a promising strategy to obtain new interstocks/rootstocks with improved *C*Las/CTV resistance/tolerance and better horticultural performance.

It is interesting to note that another Oceanian genotype, ARLxLLA-FDC6, was able to partially impair *C*Las movement to the roots in both experiments, but the percentage of trees without *C*Las detection in the root system varied between evaluations. This may be related to physiological, ontological and/or environmental conditions that may affect the genetic resistance mechanisms at the whole plant level. Considering also that all Oceanian genotypes evaluated in this work were previously selected as completely free of *C*Las infection in the leaves/shoots (Alves et al., 2021b), but there was a variation of *C*Las infection when tested as interstocks, our results suggest that the HLB resistance outcome may vary considerably among Oceanian genotypes and/or within plant tissues, being likely affected by quantitative x environment traits. Alternatively, *C*Las may have been translocated from the scions to the rootstocks just passively, through the flux sap, as photoassimilates are moved from source leaves to sink roots (Raiol-Junior et al., 2021). In this sense, we investigated the sieve plate of phloem cells of *C*Las-inoculated interstock barks by SEM in experiment I. No anatomical damages or plugging of the pores were observed in the phloem of ARLxLLA-FDC6 and AFL-BGC695 (which were the interstocks leading to lower *C*Las infection in the rootstocks), while the phloem cells of VSWO and ADL-FDC12 (which were the interstocks leading to higher infection rates) presented plugging of the pores. These results suggest that the former genotypes are less susceptible to the disease without limiting the phloem sap flow in the plant. The ratio of pore surface area to cell surface area in *C*Las-positive and *C*Las-negative samples was also evaluated, and the decrease of the ratios in *C*Las-positive samples compared to the *C*Las-negative ones in most genotypes may be interpreted as a collapse of the tissue structure (limited change of the pore area but an increase of the cell surface). Interestingly, VSWO controls showed the highest decrease of such ratio and much larger phloem pores than Oceanian genotypes that presented higher *C*Las blockage. Therefore, these anatomical features may play a role in the HLB resistance phenotype of Oceanian limes. Nevertheless, the impact of CTV on the anatomical disturbance of phloem cells cannot be ruled out in the case of these genotypes (Khalilzadeh et al., 2024, 2025).

To provide further insights into HLB responses induced by the interstocks on the VSWO scion, some biochemical and molecular analyses were performed. As expected, total polyphenol contents in the leaves of *C*Las-infected trees on the interstock control (VSWO) were higher than those observed in *C*Las-free plants, as previously observed in other susceptible genotypes (Sivager et al., 2022). However, no substantial changes in the leaf polyphenol contents were observed on VSWO scions using any of the Oceanian interstocks evaluated, suggesting that the bacterium did not induce oxidative stress that favors the production of polyphenols in these genotypes (Martinelli et al., 2016). These results reinforce that Oceanian citrus interstocks may have remained uninfected or only slightly infected by the bacterium in their phloem cells, while *C*Las was able to move mostly passively through the sap flux. Moreover, we selected target genes related to plant defense to evaluate their relative expression (RE) levels in *C*Las-infected versus control trees grafted onto the different interstock genotypes, and compared them to healthy VSWO trees as the standard. It is well known that pathogen infection results in the activation of plant defense mechanisms and modulation of sets of genes that may encode antimicrobial proteins (Thomma et al., 1998; Durrant and Dong, 2004). In this study, the use of VSWO scion leaves for RE analysis aimed to capture potential shifts in defense gene expression in the HLB-susceptible scion, but RE responses to *C*Las in specific tissues of the most promising Oceanian genotypes, like ARLxLLA-FDC6 and AFL-BGC682, remain to be investigated. Our data showed that the different interstocks influenced the upregulation of defense genes, but *C*Las infection vs. no infection was the most important variable inducing contrasting defense gene upregulation of most of the target genes evaluated. This was remarkably evident for *Pathogenesis Related Protein 2* (*PR-2*), the plant-specific transcription factors (*WRKY70* and *WRKY25*), and a gene of disease resistance family protein (*RLP7*). Increased accumulation of pathogenesis-related (PR) proteins, such as *PR-2*, a pathogen-inducible gene, has been reported in HLB x *Citrus* pathosystems using two different rootstocks and exogenous application of systemic acquired resistance (SAR) inducers (Widyawan et al., 2023). These authors also observed differential responses on the plant scion, even though the effective contribution of *PR-2* in the HLB pathosystem remains unknown. *WRKY* transcription factors can control various reactions via a complex gene network, providing simultaneous multiple responses upon pathogen challenge and infection (Phukan et al., 2016). Moreover, in our study, Oceanian interstocks induced upregulation of some defense gene homologs in *C*Las-free compared to control VSWO plants. It is known that different genotypes evaluated at the rootstock position can influence the overall gene expression of the scion variety subjected to different stresses in citrus trees (Hu et al., 2022; Liu et al., 2017). However, the response of sweet orange to single infection by either *C*Las or CTV has been well characterized by global gene expression analyses (Gandía et al., 2007; Fan et al., 2011), and Fu et al. (2016) observed that the expression of transcription factors, including *WRKY*s, occurs in response to co-infection with *C*Las and CTV. Thereupon, the presence of CTV in scions and rootstocks, as well as its multiplication, especially in most of the Oceanian interstocks used, may explain at least in part the de-regulation of such defense gene markers, though it requires further investigation.

Interstocks have been mainly used to overcome graft incompatibility issues between specific scion and rootstock varieties and gained commercial relevance in recent years in the Brazilian citriculture, with millions of ‘Pera’ sweet orange scions being grafted onto ‘Valencia’ interstocks and ‘Swingle’ citrumelo rootstocks (Carvalho et al., 2019; Girardi et al., 2021). Although field experiments evaluating different graft combinations with interstocks reported some improvement in HLB tolerance, most trees were ubiquitously infected with *C*Las because the citrus genotypes evaluated were all susceptible to bacterial colonization (Shokrollah et al., 2011; Dutt et al., 2023). In this work, we evaluated Oceanian HLB-resistant genotypes as interstocks between HLB-susceptible ‘Valencia’ sweet orange scion and ‘Rangpur’ lime rootstock, and our results indicate that this propagation technique was unable to either completely prevent *C*Las movement from the graft-infected scion into the rootstock or decrease *C*Las detection frequency and titers in the scion of trees under greenhouse conditions. Therefore, the interstocks tested here have a limited potential in HLB management in the short term. However, the two Oceanian hybrid genotypes used were able to partially block the long-distance movement of *C*Las from scions to rootstocks. In these specific cases, it remains to be tested if larger interstock segments or top-working may restrict further *C*Las movement from the scion to the rootstock. Co-infection of *C*Las with CTV was a pre-requisite in our experimental approach because CTV inoculation to induce cross-protection is mandatory to avoid aggressive CTV symptoms as part of the citrus budwood propagation in nurseries of São Paulo State (Brazil. In general, the effect of CTV infection and multiplication resulted in poor growth of interstocked trees regardless of *C*Las infection, even though there was an additional decrease in the root weight of plants with *C*Las-infected scions. This result alerts to a major drawback for the use of certain Australian citrus species and hybrids for HLB resistance breeding purposes, as they may be resistant to *C*Las multiplication but at the same time could be susceptible to CTV, precluding practical uses in citrus regions where CTV is endemic. Studies using true-to-type *C. glauca* and other CTV-tolerant Australian citrus types as interstock varieties may be necessary to attempt complete blockage of *C*Las movement from scion to rootstock, conjugated to better composite plant development. In this way, some of the crosses already generated of *C. australis*, *C. inodora*, and *C. glauca* with CTV-tolerant Asian citrus genotypes may lead to more effective disease control in the future, in addition to increased genetic diversity for citrus orchards.

## Data Availability

The original contributions presented in the study are included in the article/**Supplementary Material**. Further inquiries can be directed to the corresponding author.
