# Supplementary material for: Can an HLB-resistant interstock block the long-distance movement of ‘Candidatus Liberibacter asiaticus’ within citrus trees?
Source: Front Plant Sci. 2026 Mar 25;17:1733981. doi: 10.3389/fpls.2026.1733981 (PMC13057377; doi:10.3389/fpls.2026.1733981)
Supplement: Supplementary file 2 [file SupplementaryFile1.docx]

**
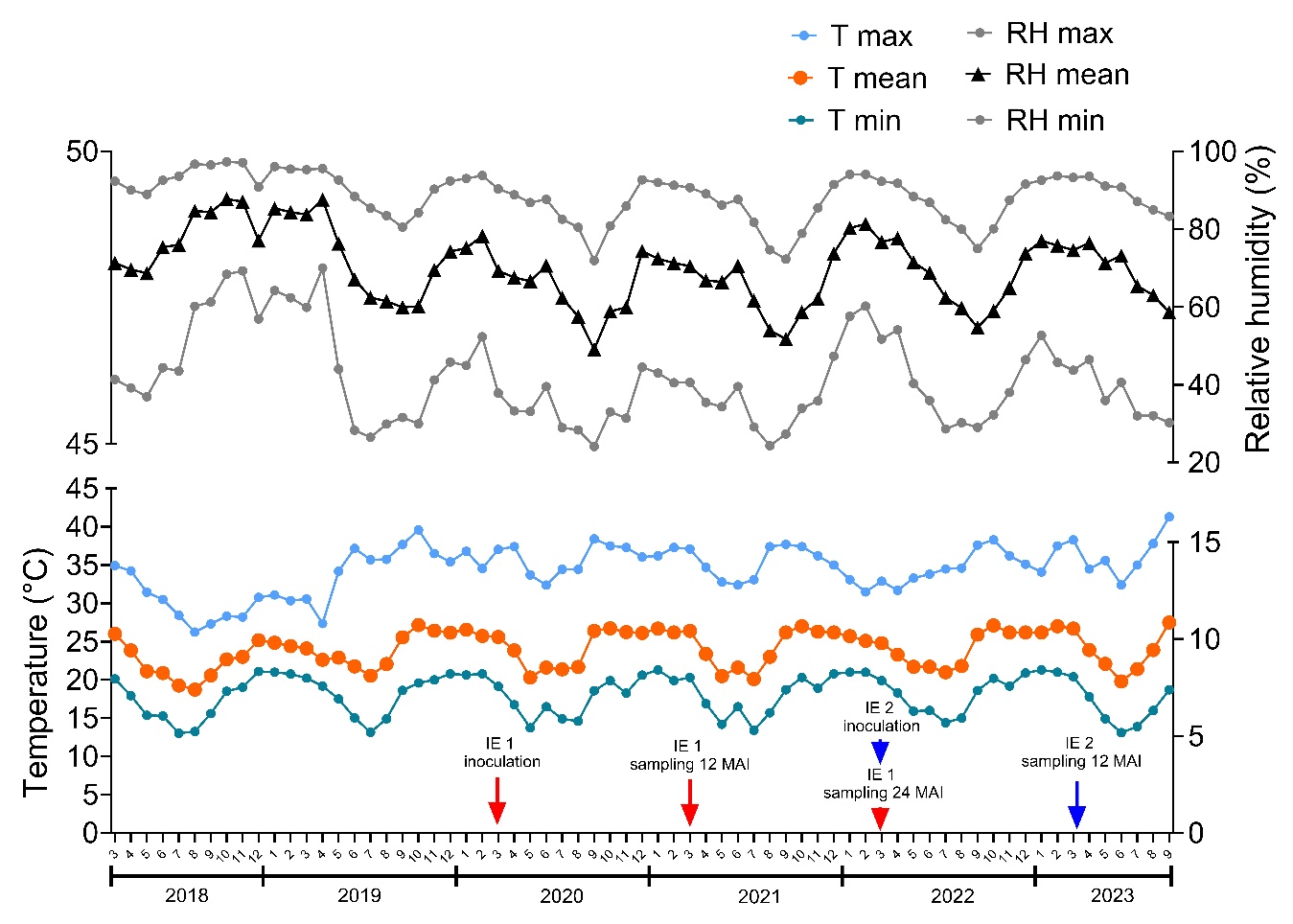
**

**Supplementary Figure 1.** Environmental conditions and experimental timeline during the experiments interstock I (IE 1) and II (IE 2) (2018–2023).


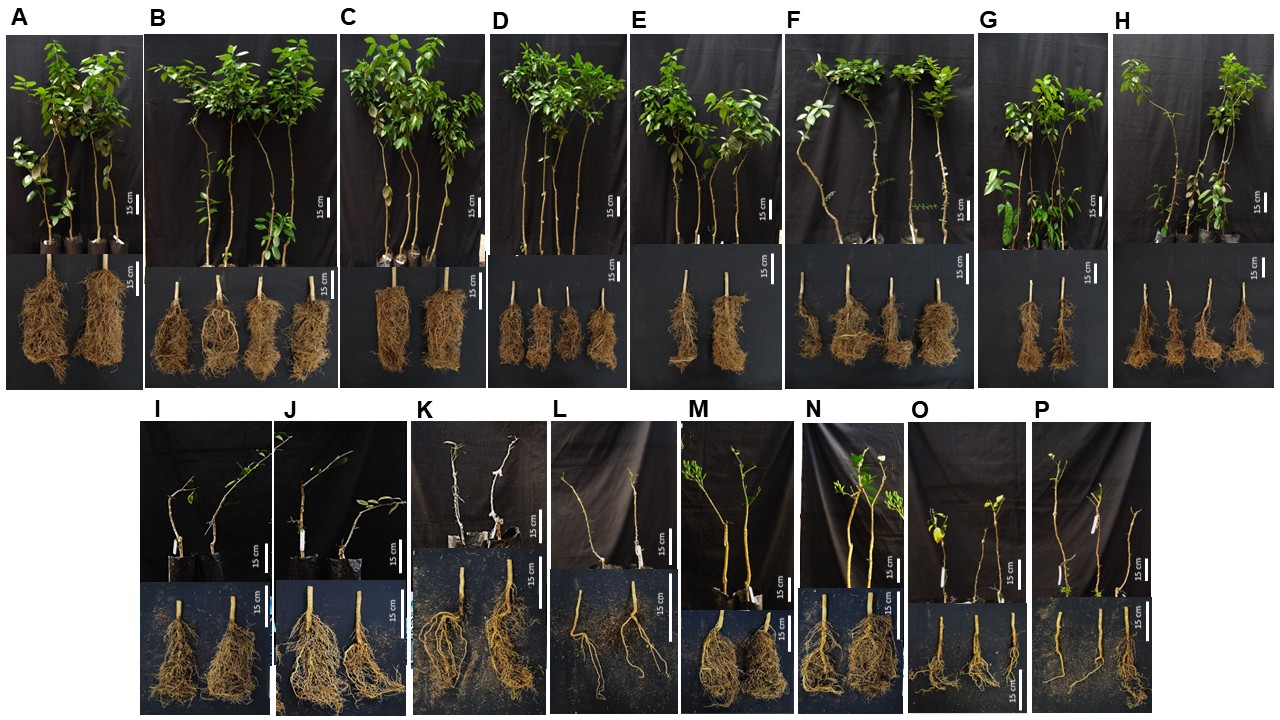


**Supplementary Figure 2.** -Visual assessment of plant growth and root architecture. VSWO (*Citrus* × *sinensis* var. ‘Valencia’) healthy (**A**) and *C*Las-infected (**B**); ADL-FDC12 healthy (**C**) and *C*Las-infected (**D**); AFL-BGC695 healthy (**E**) and *C*Las-infected (**F**); ARLxLLA-FDC6 healthy (**G**) and *C*Las-infected; (**H**); BRFLxNWL-FDC2 healthy (**I**) and *C*Las-infected (**J**); ADL-BGC682 healthy (**K**) and *C*Las-infected (**L**); ADL-BGC682 healthy (**M**) and *C*Las-infected (**N**); NGL-FDC7 healthy (**O**) and *C*Las-infected (**P**), at the termination of the experiment II. Genotypes details in table 1.


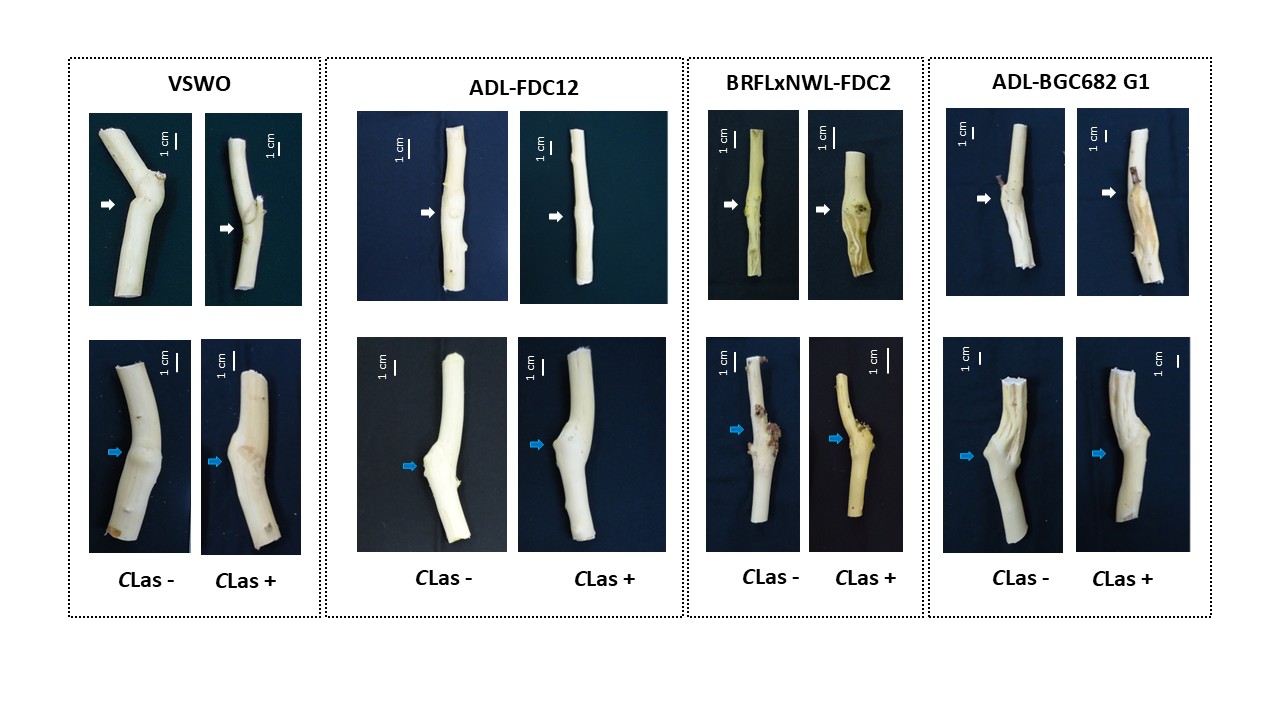


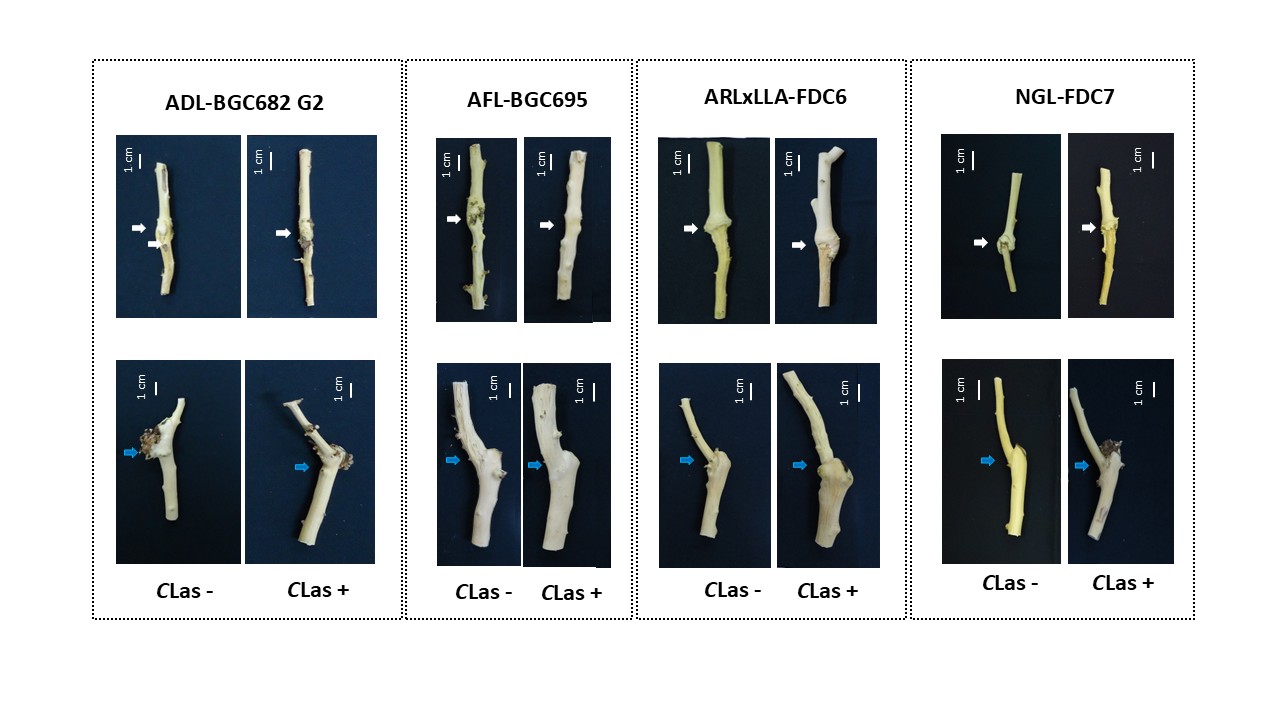


**Supplementary Figure 3.** Visual assessment of graft compatibility and stem pitting symptoms in stems of healthy or *C*Las-infected plants of ‘Valencia’ sweet orange scion grafted onto ‘Rangpur’ lime rootstock using interstocks of *Citrus* × *aurantium* L. var. sinensis ‘Valencia’ (VSWO); hybrid ADL-FDC12; hybrid BRFLxNWL-FDC2; hybrid ADL-BGC682 (G1) and ADL-BGC682 (G2); hybrid AFL-BGC695; hybrid ARL-LLA-FDC6); and true-to-type *C. warburgiana* F.M. Bailey (NGL-FDC7). The blue arrow shows the bud union at the scion/interstock and white interstock/rootstock positions, respectively, at the termination of the experiment II. Genotypes details in table 1.
